# Tanscriptomic Study of the Soybean-*Fusarium virguliforme* Interaction Revealed a Novel Ankyrin-Repeat Containing Defense Gene, Expression of Whose during Infection Led to Enhanced Resistance to the Fungal Pathogen in Transgenic Soybean Plants

**DOI:** 10.1371/journal.pone.0163106

**Published:** 2016-10-19

**Authors:** Micheline N. Ngaki, Bing Wang, Binod B. Sahu, Subodh K. Srivastava, Mohammad S. Farooqi, Sekhar Kambakam, Sivakumar Swaminathan, Madan K. Bhattacharyya

**Affiliations:** Department of Agronomy, Iowa State University, Ames, IA, United States of America; Chinese University of Hong Kong, HONG KONG

## Abstract

*Fusarium virguliforme* causes the serious disease sudden death syndrome (SDS) in soybean. Host resistance to this pathogen is partial and is encoded by a large number of quantitative trait loci, each conditioning small effects. Breeding SDS resistance is therefore challenging and identification of single-gene encoded novel resistance mechanisms is becoming a priority to fight this devastating this fungal pathogen. In this transcriptomic study we identified a few putative soybean defense genes, expression of which is suppressed during *F*. *virguliforme* infection. The *F*. *virguliforme* infection-suppressed genes were broadly classified into four major classes. The steady state transcript levels of many of these genes were suppressed to undetectable levels immediately following *F*. *virguliforme* infection. One of these classes contains two novel genes encoding ankyrin repeat-containing proteins. Expression of one of these genes, *GmARP1*, during *F*. *virguliforme* infection enhances SDS resistance among the transgenic soybean plants. Our data suggest that *GmARP1* is a novel defense gene and the pathogen presumably suppress its expression to establish compatible interaction.

## Introduction

Soybean [*Glycine max* (L.) Merr.] is an economically important crop. Sudden death syndrome (SDS) is one of the most serious soybean diseases and a major cause of soybean yield losses in the United States as well as in South American countries [1–3]. In North America, it is caused by the soil-borne fungus, *Fusarium virguliforme* O’Donnell and T. Aoki (formerly *F*. *solani* (Mart.) Sacc. f. sp. *glycines*); whereas in South America, it is caused by four *Fusarium* spp., *F*. *virguliforme*, *F*. *tucumaniae*, *F*. *brasiliense*, and *F*. *cuneirostrum* [4,5]. Of the four *F*. *spp*., *F*. *tucumaniae* is the major causal agent of SDS in South America [4]. *F*. *virguliforme* is asexually propagated, whereas *F*. *tucumanie* is sexually propagated. Recently, it has been shown that *F*. *tucumaniae* carries two idiomorphs at the *MAT* locus, whereas *F*. *virguliforme* carries only one [6].

*F*. *virguliforme* is a hemi-biotrophic fungus that remains in soil. It attacks roots and produces root rot symptoms [7,8]. The pathogen has never been detected in the aboveground diseased plants. In infected roots, it produces fungal toxins including FvTox1 that cause foliar SDS [9–13]. Additional candidate toxins have been detected in xylem sap of *F*. *virguliforme*-infected soybean plants [14].

Gene expression profiling using RNA sequencing has facilitated understanding the molecular basis of plant-pathogen interactions. Such studies have revealed interesting novel genes and pathways modulated following pathogen infections, and their myriad of responses to overcome pathogen attacks [15–18].

Transcriptome profiles of soybean and other crops infected with pathogens have brought new insights in our understanding of host-pathogen interactions. For instance, Moy and colleagues [19] reported that defense and pathogenesis-related protein genes were strongly induced while lipoxygenases and peroxidases genes were strongly repressed during infection of soybean with the oomycete pathogen *Phytophthora sojae*. Following inoculation of soybean with *F*. *virguliforme*, many defense-related genes were up-regulated in the partially resistant soybean recombinant inbred line 23 (RIL23), whereas these genes were either unchanged or down-regulated in the SDS susceptible cultivar, ‘Essex’ [20]. Defense-related genes have been shown to be induced in both resistant and susceptible soybean cultivars following *F*. *virguliforme* infection [21].

In the US, although SDS was first detected in Arkansas only in 1971, it has now spread throughout the soybean growing areas of the North Central United States and Canada [22–25] and is becoming a serious threat to soybean production. The disease has been reported to cause soybean yield losses valued over 100 million dollars [2]. Options for managing SDS are limited. Use of resistant cultivars has been the most effective method of managing this disease. Unfortunately, SDS resistance is partial and governed by a large number of QTL, each contributing a small effect [26–29]. To date, more than 40 QTL for SDS resistance have been reported [30]. As a result, development of SDS resistant soybean lines by combining a large number of QTL by hybridization is not trivial; and therefore, identification of novel single major genes conferring SDS resistance is becoming essential. Unfortunately, it is very unlikely that there are any natural major genes in managing this emerging disease problem. Therefore, development of transgenic soybean lines with manipulated expression of candidate or known defense genes is becoming very urgent for controlling SDS. Earlier it has been demonstrated that transgenic approaches can effectively reduce the yield losses caused by pathogens [31–34].

We hypothesize that pathogens suppress defense-related genes to overcome potent host defense mechanisms to establish in host cells, multiply and spread. To our knowledge, no attempt has been made to alter the expression of down-regulated putative host defense genes to enhance disease resistance in transgenic plants. This study was undertaken primarily to uncover candidate defense-related genes repressed during *F*. *virguliforme* infection and to determine if altered expression of such a gene can enhance resistance against *F*. *virguliforme*. We examined the expression profile of soybean genes in roots of young etiolated seedlings infected with *F*. *virguliforme* conidial spore suspensions or treated with sterile water. We observed that following inoculation with *F*. *virguliforme*, transcripts of more genes were up-regulated than down-regulated. We altered the expression of one member, of a family of two down-regulated genes, *GmARP1* and *GmARP2*, encoding ankyrin repeat-containing proteins during *F*. *virguliforme* infection in transgenic soybean plants. Several independent transgenic soybean plants showing induced expression of *GmARP1* exhibited enhanced SDS resistance. Our study suggests that (i) *F*. *virguliforme* somehow suppresses defense-related genes to cause susceptibility and (ii) *GmARP1* encoding ankyrin repeats containing protein is a defense gene.

## Materials and Methods

### Plant materials, treatments, and growth conditions

For RNA-sequencing (RNA-seq) experiment, soybean seeds of cultivar ‘Williams 82’ were sown in vermiculite and grown under the dark for 10 days according to Bhattacharyya and Ward [35]. Etiolated seedlings were inoculated with either water (water treatment) or *F*. *virguliforme* conidial spores (infection) at a concentration of 10^7^ spores ml^-1^. Root samples were harvested at different time-points, 3, 5, 10 and 24 days following inoculation with *F*. *virguliforme* or treatment with sterile water [10]. We grouped the samples in four categories: S1, a pooled sample of equal amounts RNAs isolated from roots collected 3 and 5 days following water treatment; S2, a pooled sample of equal amounts RNAs isolated from roots collected 10 and 24 days following water treatment; S3, a pooled sample of equal amounts RNAs isolated from roots collected 3 and 5 days following inoculation with *F*. *virguliforme*; S4, a pooled sample of equal amounts RNAs isolated from roots collected 10 and 24 days following inoculation with *F*. *virguliforme*. For three independent RT-PCR experiments, RNA samples were prepared from roots harvested 8 h, 12 h, 1 d, 3 d, and 5 d following either treatment with water or inoculation with *F*. *virguliforme*.

### DNA isolation, plasmid vector construction, and soybean transformation

Three root specific and infection inducible promoters (Prom) and the *GmARP1* gene (S1 and S2 Figs) were amplified from soybean cv. Williams 82 DNA. Prom 1 (*Glyma18g47390*) was discovered in our lab (B.B. Sahu and M.K. Bhattacharyya, unpublished); Prom 2 (*Glyma10g31210*) and Prom 3 (*Glyma20g36300*) are two root specific promoters, reported earlier (http://www.oardc.ohio-state.edu/SURE/GmROOT/GmRoot.htm). Genomic DNA was isolated using a modified CTAB extraction method [36] adapted from Doyle and Doyle [37]. Promoter sequences were amplified using the following pairs of primers: *Prom1F*-*Prom1R*; *Prom2F*-*Prom2R*; *Prom3F*-*Prom3R* (S1 Table). The sequence of *GmARP1* was amplified using the primers GmARP1G-F and GmARP1G-R. In these primers, sequences in bold font indicate cloning sites. The binary vector pTF102 [38] (S3 Fig) was used to create three *GmARP1* transgenes: *Prom1-GmARP1*, *Prom2-GmARP1*, and *Prom3-GmARP1* as follows. First, the CaMV 35S promoter was removed from pTF102 by digesting with *Xba*I and replaced it with any of the three new promoters. The restriction site for cloning *GmARP1* (*Bst*XI) was inserted at the 3’-end of the promoter primers. Next, we excised the *GUS* gene and CaMV 35S terminator (containing the Poly(A+) signal) by digesting with *Bst*XI and the *Hind*III. The CaMV 35S terminator was reinserted with addition of the *Bst*XI restriction site at the 5’-end-specific PCR primer, and cloned in the *Bst*XI and *Hind*III sites. Finally, the created vectors were digested with *Bst*XI and the *GmARP1* sequence including 84 nucleotides upstream of the ATG start codon and 45 nucleotides beyond the TAA stop codon was inserted. The constructs were cloned in to *Escherichia coli* strain DH10B and sequenced to confirm their identity. The constructs were then transferred by electroporation into *Agrobacterium tumefaciens* strain EH101 for transformation of Williams 82 at the Plant Transformation Facility, Iowa State University. R_0_ transgenic soybean plants carrying *GmARP1* transgenes were maintained in a greenhouse. R_1_ seeds were harvested for further characterization in growth chambers and then under field conditions.

### Infection assays of transgenic soybean plants

#### Evaluation of transgenic plants in growth chambers

For inoculation of transgenic soybean plants, *F*. *virguliforme* Mont-1 was grown on 1/3 potato dextrose agar (PDA) plates for three to five weeks. We prepared the inocula on sorghum grains and mixed with a 1:1 mixture of sand and soil in a 1:20 inoculum: soil ratio for sowing soybean seeds [39]. To assess the responses of the R_1_ progenies to *F*. *virguliforme* infection, we conducted three independent inoculation experiments as follows. We evaluated responses of 15 to 30 R_1_ progenies to *F*. *virguliforme* infection by sowing 3 seeds in a 237-ml Styrofoam cup containing the inocula mixed soil and sand mixture. The cups were then placed in growth chamber maintained at 22–23°C and 16 h light and 8 h dark. The light intensity was 350 μE/m^2^/s. The plants were watered daily.

Foliar symptoms were scored 4 weeks following planting in a 1 to 7 scale, modified from previously published protocols [40–42]. Plants were considered resistant if they showed symptoms of scores 1 and 2 with symptoms of slight yellowing. The plants were classified as susceptible when disease scores were 3 to 7 characterized by severe chlorosis to necrosis. For molecular analysis, roots of infected plants were harvested and frozen in liquid nitrogen. Chlorophyll contents in leaves of infected plants were used as a measure of foliar symptoms. Extraction and estimation of chlorophyll contents were conducted according to [43]. Extent of root rots was visually evaluated and root resistance to the pathogen was calculated in percentage of healthy roots with no obvious blackening caused by necrosis and rotting.

#### Field evaluation of transgenic plants with SDS pathogen

A field test of transgenic soybean plants was carried out in the Hinds Research Farm, Iowa State University located in north of Ames, Iowa between June 11 and October 30, 2015. Each transgenic line carrying *GmARP1* transgenes were grown in two replications along with the SDS resistant cultivar, MN1606, and the SDS susceptible transgene recipient line, Williams 82. Seeds of individual genotypes were mixed with *F*. *virguliforme* NE305S inoculum grown on sorghum grains during planting with a push planter. At 1 to 2-trifoliate stage, all transgenic lines were sprayed with basta herbicide (glufosinate at a 250 mg/L concentration) mixed with 0.1% Tween 20 twice with an interval of two days (S2 Table). DNA samples were harvested from twelve plants that showed resistance to the herbicide. The plants were heavily irrigated in the last week of August that followed by heavy rains. SDS symptoms appeared following heavy rainfall and flood. Individual plants were scored on September 11^th^, 22^nd^, 30^th^, and October 7^th^, based on a scale of 1 to 9, with 1 being symptomless to 9 for severe symptoms with death of soybean plants (S3 Table) (www.siu.edu/~soybean).

### RNA extraction, RNA sequencing, sequence assembly and alignment of reads to *Glycine max* reference genome

Total RNA samples were extracted from root tissues using the SV Total RNA isolation system (Promega, Madison, WI, USA) following the protocol provided by the manufacturer. The amount and the quality of RNAs in each sample were determined using a spectrophotometer and running on formaldehyde agarose gels, respectively. RNA sequencing was conducted on an Illumina HiSeq 2500 instrument at the DNA Facility, Iowa State University. The sequences were first processed for quality check using FASTX tool-kit. They were then indexed on the soybean reference genome using the open source Bowtie 2 tool [44]. The processed files were aligned to corresponding predicted high confidence coding sequences of the *Glycine max* reference genome to calculate RPKM values using Bowtie program and generated SAM (Sequence Alignment/Map) output files for each condition using unix script command [45]. For GO annotation, sequences of differentially expressed genes (DEG) were extracted from Soyabase.org through scripts and Phytozome [46]. The assigned biological function to the DEG was categorized further based on their molecular functions, biological processes and cellular component.

### Semi-quantitative RT-PCR amplification

cDNA synthesis was conducted using the M-MLV reverse transcriptase following the instructions of the manufacturer from two μg of total RNAs in each sample (Promega, Inc., Madison, WI, USA). Approximately 200 to 500 bp cDNA fragments were amplified by PCR using gene specific primers for five soybean genes. PCR was conducted for 25 cycles using the following condition: Step 1, 94°C for 2 min; Step 2 94°C for 30 sec; Step 3, at annealing temperature of 60°C for 30 sec; Step 4, extension for 1 min at 72°C; Step 5, repeated cycles 2 through 4 for 24 more times; Step 6, final extension step of 10 min at 72°C. For the three independent RT-PCR experiments of Fusarium-infected and water-treated roots, gene specific primers of each of the four selected genes were used to determine their transcript levels in infected and non-infected roots (S4 Table). Expression of soybean levels was quantified by analyzing the scanned gels carrying electrophoresed RT-PCR products with the ImageJ program (http://imagej.nih.gov/ij/) [47].

For expression analysis of *GmARP1* transgenes among transgenic plants, *GmARP1*-specific forward (GmARP1-RT-F) and reverse primer specific to the poly(A+) signal of transgenes (RT-pTF102-R) were used to determine the expression levels of the *GmARP1* transgenes (S1 Table).

### Transgene copy number analysis by qPCR

Genomic DNA was extracted from young leaves of 12 transgenic plants for each line. We used approximately 50 mg of lyophilized leaf tissues for DNA extraction at the Iowa State University DNA Facility using the fully automated system, Autogen Autogenprep 740 DNA extraction robot (AutoGen, MA, USA). DNA quantity in each sample was determined using a nanodrop spectrophotometer, and diluted to 20 ng per μl for qPCR reaction.

qPCR was conducted on a Biomark HD system using the 192.24 Taqman CNV protocol (Fluidigm, South San Francisco, CA, USA). Two Taqman assays were designed, the *bar* gene (target) and the reference gene (an endogenous single copy gene, *Glyma*.*05G014200*). Reporter/quencher dyes were FAM/MGB-NFQ for *bar* and VIC/TAMRA for the reference gene. Data were analyzed using a Biomark HD data collection software and the copy number for the *bar* gene was calculated.

## Results

### Identification of differentially expressed soybean genes following *F*. *virguliforme* infection

Ten day-old seedlings of cultivar Williams 82 were either treated with water (water treatment) or infected with *F*. *virguliforme* isolates. In order to monitor the expression of genes during infection, roots tissues were harvested at different time periods: (i) S1, early time period (ETP) of pooled root samples, 3 and 5 days following water treatment; (ii) S2, late time period (LTP) of pooled root samples, 10 and 25 days following water treatment; (iii) S3, ETP of pooled root samples, 3 and 5 days following *F*. *virguliforme* infection; (iv) S4, LTP of pooled root samples, 10 and 24 days following *F*. *virguliforme* infection. Total RNA samples were extracted from the root tissues and sequenced using Illumina HiSeq 2500 (Illumina, San Diego, CA) and deposited in GEO (accession GSE86201).

The deep-transcript sequencing experiment was conducted only once. We therefore, considered the genes showing at least 10-fold or more changes in transcript levels between infected and control tissues as the differentially expressed genes (DEGs). Furthermore, we considered only those genes as DEGs that have shown to contain at least five sequence reads or fragments per kilobase pair exon sequences in at least one of the treatments considered for comparison. RPKM (reads per kilobase of exon model per million mapped reads) values for individual genes were calculated to normalize the expression levels of individual genes and were used in calculating the fold changes. To validate the transcriptomic data, we conducted three independent biological replications of an RT-PCR experiment for four soybean genes that were repressed following *F*. *virguliforme* infection.

**Fig 1:**
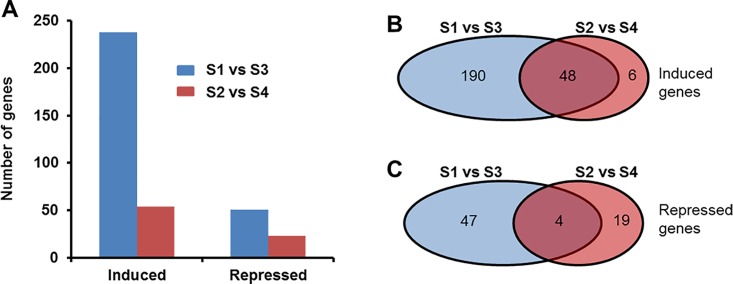
Distribution of differentially expressed genes in soybean roots in response to *F*. *virguliforme* infection. A. Total number of genes differentially (with FC ≥ 10) regulated by *F*. *virguliforme* infection. B. Number of genes up-regulated in the infected roots at early and late time-periods. C. Number of genes repressed in the infected roots at early and late time-periods. S1, pooled RNA samples prepared from roots harvested 3 and 5 days following water treatment; S2, pooled RNA samples prepared from roots harvested 10 and 24 days following water treatment; S3, pooled RNA samples prepared from roots harvested 3 and 5 days following *F*. *virguliforme* infection; S4, pooled RNA samples prepared from roots harvested 10 and 24 days following *F*. *virguliforme* infection.

Pairwise comparison of the expression levels of soybean genes in inoculated roots with those of corresponding water treated roots during ETP- or LTP revealed 314 DEGs that showed ≥ 10-fold change (FC). In identifying DEGs, we considered only those genes that showed to contain at least 5 sequence reads per kilobase pair exon sequences in at least one of the treatments considered for comparison. We detected transcripts for 54,305 of the predicted soybean genes [48,49]. We found more DIGs in roots of ETP than that in LTP (Fig 1A). In infected roots of both ETP and LTP, there were more up-regulated genes than the down-regulated ones (Fig 1). During ETP, 289 genes were differentially expressed between infected and water-treated roots with FC ≥ 10. The majority of these DIGs (238; 82%) were induced; only 54 genes (18%) were repressed in the infected roots of ETP as compared to the water treated root tissues (Fig 1B and 1C; Table 1; S1 and S2 Datasets). In the infected root tissues of LTP, of the 77 DIGs, 54 (70%) were up-regulated and 23 (30%) were down-regulated (Fig 1B and 1C; Table 2, S3 Dataset).

**Table 1:** Genes down-regulated (with FC >10) in soybean roots during early time period following infection with *F*. *virguliforme*. The full list is reported in S2 Dataset.

Locus ID	RPKM		Fold change	P-value	Functional Annotation
	S1	S3			
Glyma.01g171600	12.38	0.05	254.8	1.12E-06	SAM dependent carboxyl methyltransferase
Glyma.02g054200	37.71	0.17	225.5	9.33E-15	SAM dependent carboxyl methyltransferase
Glyma.07g098500	19.19	0.13	144.4	6.39E-08	copper transport protein atox1-related
Glyma.08g163600	1.29	0.02	74.4	2.13E-04	pre-mrna processing protein prp39-related
Glyma.09g022800	193.8	2.63	73.7	7.33E-15	peroxidase
Glyma.10g185900	8.01	0.11	71.5	2.34E-09	sieve element occlusion protein
Glyma.03g024300	1199.19	17.48	68.6	2.51E-06	glycosyl hydrolases family 18
Glyma.03g024200	1496.7	22.9	65.4	3.13E-05	glycosyl hydrolases family 18
Glyma.03g024400	1102.06	16.91	65.2	3.53E-07	glycosyl hydrolases family 18
Glyma12g12470*	10.06	0.16	62.1	3.05E-10	ankyrin repeat-containing
Glyma.10g266500	1.34	0.02	60.5	4.50E-04	plant protein of unknown function
Glyma03g02810*	56.25	1.01	55.8	1.27E-13	glycoside hydrolase
Glyma.03g025000	53.91	0.97	55.5	3.73E-13	glycosyl hydrolases family 18
Glyma03g02818*	32.43	0.58	55.5	5.20E-13	unknown
Glyma03g02843*	45.41	0.82	55.2	1.66E-13	unknown
Glyma.06g294400	7.77	0.16	47.7	1.73E-09	ankyrin repeat-containing
Glyma.13g113100	11.65	0.27	43.8	6.61E-10	flavin-containing monooxygenase
Glyma.03g024800	64.4	1.53	42	1.09E-11	glycosyl hydrolases family 18
Glyma.07g204900	6.09	0.16	39	6.94E-07	lipase (class 3); alpha/beta-hydrolases superfamily protein
Glyma.14g032000	13.31	0.34	38.7	1.23E-04	unknown
Glyma.06g013200	29.27	0.82	35.7	8.37E-12	protein of unknown function (DUF2775)
Glyma.03g116300	108.09	3.21	33.7	2.86E-10	ubiquitin specific protease family C19-related
Glyma.06g300100	4.26	0.14	31.3	1.30E-05	transcription factor; MYB-related
Glyma.01g115500	40.93	1.35	30.4	3.42E-08	unknown
Glyma.10g094800	6.29	0.22	29	2.13E-05	unknown
Glyma.14g051600	18.92	0.84	22.6	2.54E-07	copper transport protein atox1-related
Glyma.13g205400	73.71	3.36	21.9	2.33E-07	AT1G11655
Glyma.14g061800	2.07	0.11	19.6	2.28E-04	HXXXD-type acyl-transferase family protein
Glyma.18g182800	6.03	0.32	18.6	5.34E-06	2-deoxyglucose-6-phosphate phosphatase 2
Glyma18g39500*	1.59	0.09	18.4	1.90E-05	NADPH oxidase; flavin adenine dinucleotide binding
Glyma14g05621*	4.93	0.3	16.3	2.87E-04	copper transport protein atox1-related
Glyma.01g115700	4.63	0.29	15.9	4.71E-06	plant protein of unknown function
Glyma.03g061200	18.63	1.2	15.5	1.60E-08	plant protein of unknown function (DUF247)
Glyma07g18225*	24.51	1.59	15.4	6.82E-06	SGNH hydrolase-type esterase superfamily protein
Glyma.11g216700	6.2	0.4	15.4	6.85E-05	unknown
Glyma.03g141900	20.64	1.37	15.1	5.40E-06	unknown
Glyma.16g197300	11.65	0.8	14.6	2.34E-04	unknown
Glyma.16g197400	11.65	0.8	14.6	2.34E-04	unknown
Glyma.08g189600	1.31	0.09	14.5	1.73E-04	lipoxygenase
Glyma.02g083500	351.22	26.37	13.3	4.78E-06	extensin
Glyma10g25800*	10.86	0.87	12.5	9.47E-06	serine-threonine protein kinase; disease resistance/LRR family
Glyma.19g144500	28.35	2.31	12.3	1.52E-04	unknown
Glyma.06g149600	4.36	0.36	12.2	2.94E-04	nodulin MtN21 /EamA-like transporter family protein
Glyma.09g023000	542.08	44.79	12.1	4.68E-04	peroxidase
Glyma0466s00200	63.01	5.4	11.7	5.39E-08	unknown
Glyma.09g099900	10.49	0.93	11.2	3.07E-05	serine/threonine protein kinase
Glyma.20g173800	13.19	1.2	11	1.25E-04	protein-tyrosine phosphatase 1
Glyma.12g225700	6.51	0.59	11	4.85E-04	unknown
Glyma.20g215500	32.66	3.02	10.8	3.71E-08	unknown
Glyma.01g002300	3.63	0.34	10.7	1.63E-04	cation transport protein
Glyma.08g285400	6.34	0.61	10.4	2.88E-05	glycosyl hydrolase family 28

S1, roots tissues 3 and 5 days following water treatment; S3, root tissues 3 and 5 days following infection.

*, Predicted genes in the old soybean genome sequence version [Glyma.Wm82.a1.v1.1 (Gmax1.01)].

**Table 2:** Genes down-regulated at late time period in soybean roots infected with *F*. *virguliforme*.

Locus ID	RPKM		Fold change	P-value	Functional Annotation
	S2	S4			
Glyma.06g300000	27.9	0.1	187.4	5.81E-05	MYB-related; myb binding domain
Glyma.02g054200	33.6	0.4	93.7	4.79E-05	SAM dependent carboxyl methyltransferase
Glyma.07g234100	187.1	3.5	53.9	1.24E-05	uncharacterized protein
Glyma.15g082200	170.5	4	42.6	8.53E-07	cysteine proteinase cathepsin F
Glyma.16g038100	943.1	23.5	40.1	4.11E-06	uncharacterized protein
Glyma13g11969*	662.7	20.4	32.5	3.43E-06	uncharacterized protein
Glyma.09g022800	93.9	3.2	29.2	6.42E-06	peroxidase
Glyma.09g163800	434.4	15.3	28.3	6.47E-06	trypsin and protease inhibitor; endopeptidase inhibitor
Glyma.03g024400	664.4	23.5	28.3	3.92E-05	hydrolase activity
Glyma.10g232100	2129.7	77.8	27.4	8.57E-05	uncharacterized protein
Glyma.06g298700	639.1	23.6	27	1.72E-05	wound-induced protein; wound-responsive
Glyma.02g303200	211.4	8.1	26	7.51E-06	uncharacterized protein
Glyma.03g024300	635.2	25.7	24.7	9.18E-05	hydrolase activity
Glyma17g03850*	32802	1367.3	24	6.10E-05	uncharacterized protein
Glyma.16g178000	262.1	11	23.8	1.21E-05	lipid-transfer/copper transport protein atox1-related
Glyma.11g224900	472.4	20.4	23.2	1.20E-05	uncharacterized protein
Glyma.17g039400	2205.5	96.7	22.8	2.26E-05	uncharacterized protein
Glyma.13g282200	117.7	5.4	21.7	2.30E-05	wound-induced protein; wound-responsive
Glyma.13g282400	82.9	3.9	21	3.73E-05	wound-induced protein; wound-responsive
Glyma13g42850*	1375.5	69.2	19.9	5.80E-05	uncharacterized protein
Glyma.12g048000	393.6	21.3	18.4	5.90E-05	uncharacterized protein
Glyma.01g210500	50.7	2.8	18.3	3.52E-05	oligopeptide transporter-related
Glyma.03g082100	197.6	13.1	15.1	9.50E-05	metallothion binding

S2, roots tissues 10 and 24 days following water treatment; S4, root tissues 10 and 24 days following infection.

*, Predicted genes in the old soybean genome sequence version [Glyma.Wm82.a1.v1.1 (Gmax1.01)].

### Functional classification of soybean genes induced in roots following *F*. *virguliforme* infection

**Fig 2:**
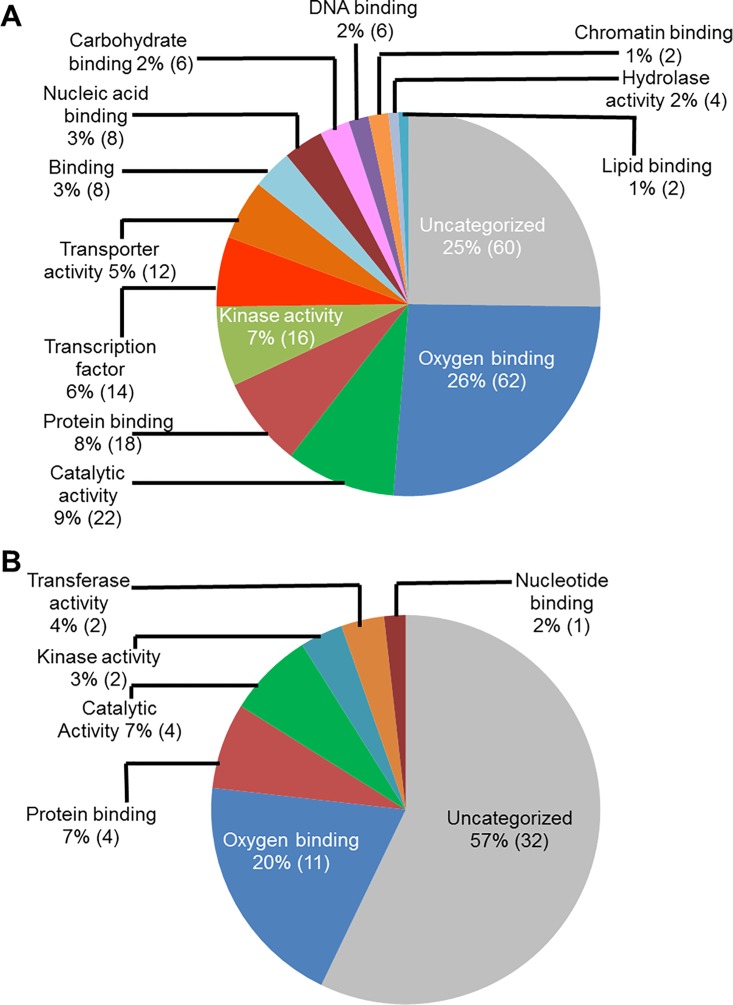
Classes of genes up-regulated in roots infected with *F*. *virguliforme* as compared to the roots treated with water. **A.** A total of 238 genes were induced (with a FC ≥10) in the pooled RNA sample of roots harvested 3 and 5 days following *F*. *virguliforme* infection as compared to the water control; and were classified based on their putative molecular functions. B. A total of 54 genes were induced in pooled RNA sample of roots harvested 10 and 25 days following *F*. *virguliforme* infection as compared to the water control; and were classified based on their putative molecular functions.

We used public transcriptomic databases such as SoyBase [50](http://soybase.org/) and Phytozome (phytozome.jgi.doe.gov), PFAM, and National Center for Biotechnology Information (NCBI) for assignment of the gene models associated with the infection-induced genes, and also for their functional annotations. Functional classification of these genes based on their putative molecular functions indicated that a majority of the genes (62 genes during ETP and 11 during LTP) had putative oxygen binding properties (Fig 2). A large number of infection-induced genes (60 genes during ETP and 32 in LTP) encode proteins with unknown molecular functions.

**Fig 3:**
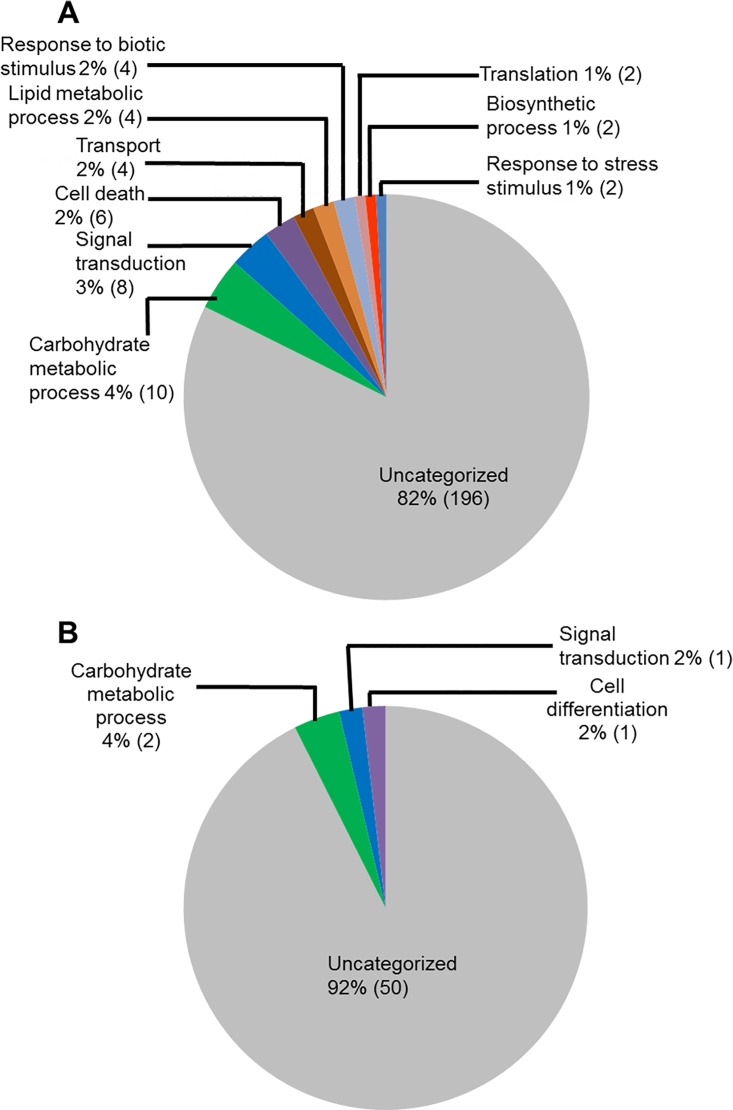
Classes of genes up-regulated in roots infected with *F*. *virguliforme* as compared to the roots treated with water. A. A total of 238 genes were induced (with a FC ≥10) in the pooled RNA sample of roots harvested 3 and 5 days following *F*. *virguliforme* infection as compared to the water control. Induced genes were classified based on their putative biological processes. B. A total of 54 genes were induced (with a FC ≥10) in pooled RNA sample of roots harvested 10 and 25 days following *F*. *virguliforme* infection as compared to the water control. Induced genes were classified based on their putative biological processes.

We investigated the infection-induced genes for their putative biological processes (Fig 3). Again a large portion of genes induced during ETP (82%) and in LTP (92%) encodes proteins with unknown functions. The next category of genes induced during ETP (Fig 3A) includes 10 genes (4%) involved in carbohydrate metabolic processes. In addition, eight genes (3%) involved in the signal transduction, six (2.5%) in cell death, four (2%) responsive to stress and stimuli, four (2%) are transporters, and four (2%) involved in the lipid metabolism processes. During LTP, only four genes (8%) were assigned with a biological process and are likely involved in the carbohydrate metabolic process, signal transduction, and cell differentiation (Fig 3B).

**Fig 4:**
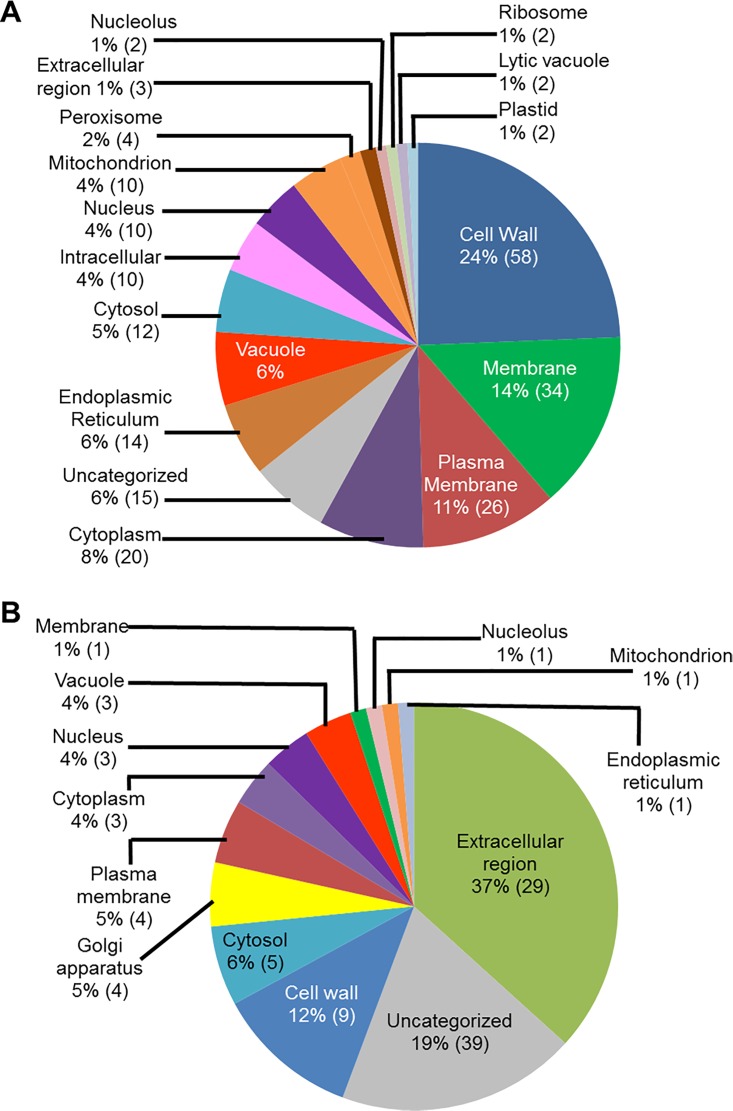
Classes of genes up-regulated in roots infected with *F*. *virguliforme* as compared to the roots treated with water. A. A total of 238 genes were induced (with a FC ≥10) in pooled RNA sample of roots harvested 3 and 5 days following *F*. *virguliforme* infection as compared to the water control. Induced genes were classified based on their putative cellular components. B. A total of 54 genes were induced (with a FC ≥10) in pooled RNA sample of roots harvested 10 and 25 days following *F*. *virguliforme* infection as compared to the water control; and were classified based on their putative cellular components.

We conducted GO analyses of the up-regulated genes for determining the putative sub-cellular locations of their encoded proteins. A large number of the genes (24%; 58 genes), up-regulated in infected-roots during ETP, most likely encode cell wall proteins (Fig 4A). Among the rest, 34 genes (14%) encode membrane bound proteins, 26 genes (11%) encode plasma membrane associated, 20 genes (8%) encode cytoplasmic proteins, and 14 genes (6%) encode endoplasmic reticulum proteins (Fig 4A). Similarly, the majority of the genes up-regulated in infected roots during LTP, 29 genes (37%) encode extracellular proteins and nine genes (12%) encode cell wall proteins (Fig 4B).

We observed that 48 of the 54 genes induced in the infected roots of LTP were induced also during ETP (Fig 1B; S1 and S3 Datasets). Functionally, 10 of the 50 genes up-regulated at both ETP and LTP belong to the cytochrome P450 CYP2 subfamily protein involved in the synthesis of defense metabolites [51]. Seven of these genes encode RmlC-like cupins with a nutrient reservoir activity; five genes encode peroxidases probably associated with signaling [52]; four genes encode receptor-like kinases (RLKs) presumably to modulate the induction of immunity [53]; three genes are members of the thaumatin family of pathogenesis-related protein that were shown to be induced during *F*. *virguliforme* infection [21], and three encode chitinase-related proteins involved in plant defense mechanisms [54,55]. Among the genes induced only in infected roots during LTP include: (i) a cupin-like gene, (ii) two defense/pathogenesis-related genes, and (iii) a gene with unknown function (S3 Dataset).

### Functional classification of soybean genes repressed in roots infected with *F*. *virguliforme*

**Fig 5:**
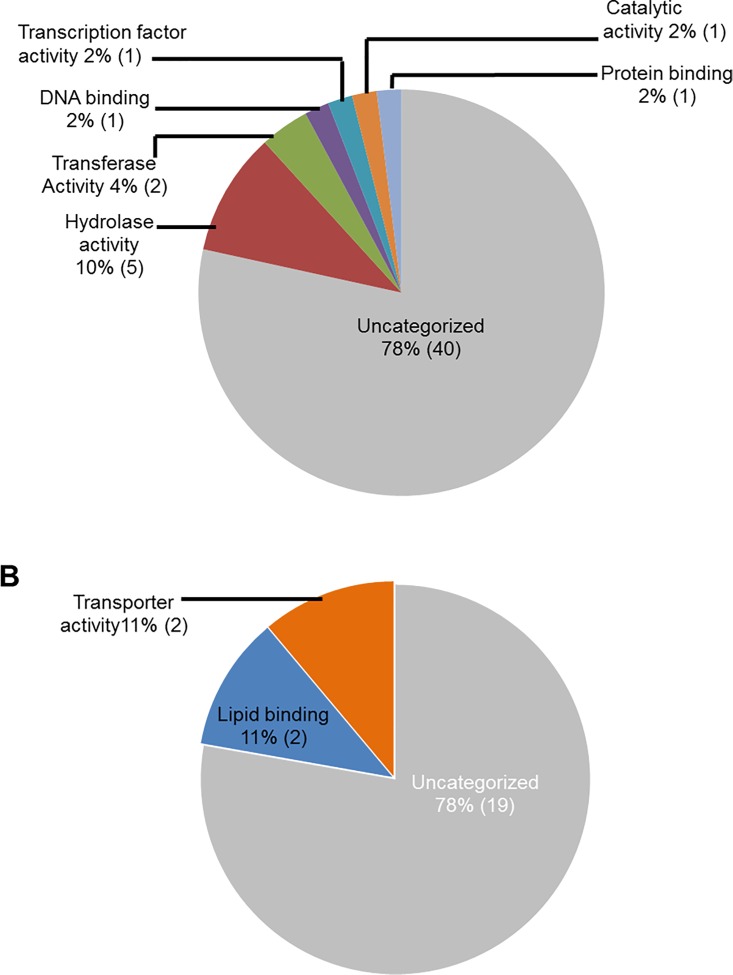
Classes of genes down-regulated in roots infected with *F*. *virguliforme* as compared to the roots treated with water. A. A total of 51 genes were repressed (with a FC ≥10) in pooled RNA sample of roots harvested 3 and 5 days following *F*. *virguliforme* infection as compared to the water control; and were classified based on their putative molecular functions. A total of 23 genes were repressed (with a FC ≥10) in pooled RNA sample of roots harvested 10 and 25 days following *F*. *virguliforme* infection as compared to the water control; and were classified based on their putative molecular functions.

**Fig 6:**
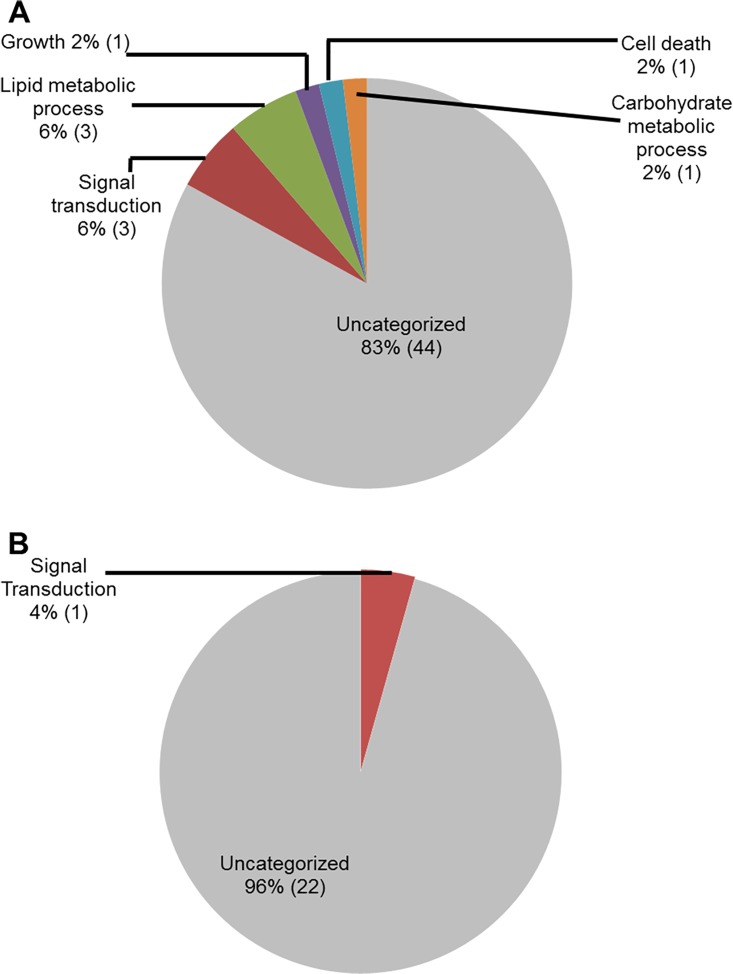
Classes of genes down-regulated in roots infected with *F*. *virguliforme* as compared to the roots treated with water. A. A total of 51 genes were repressed (with a FC ≥10) in pooled RNA sample of roots harvested 3 to 5 days following *F*. *virguliforme* infection as compared to the water control; and were classified based on their putative biological processes. B. A total of 23 genes were repressed in pooled RNA sample of roots harvested 10 and 25 days following *F*. *virguliforme* infection as compared to the water control; and were classified based on their putative biological processes.

**Fig 7:**
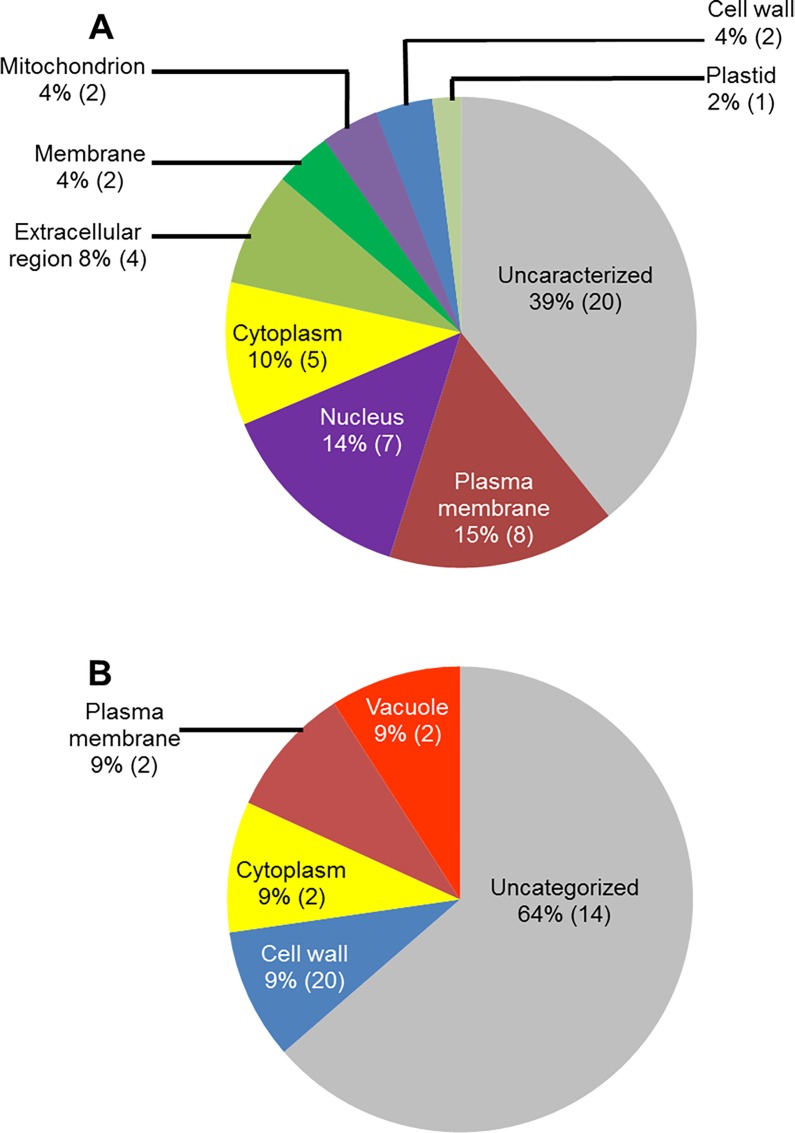
Classes of genes down-regulated in roots infected with *F*. *virguliforme* as compared to the roots treated with water. A. A total of 51 genes were repressed (with a FC ≥10) in pooled RNA sample of roots harvested 3 to 5 days following *F*. *virguliforme* infection as compared to the water control; and were classified based on their putative cellular components. B. A total of 23 genes were repressed in pooled RNA sample of roots harvested 10 and 25 days following *F*. *virguliforme* infection as compared to the water control; and were classified based on their putative cellular components.

We observed that steady-state transcript levels of 51 soybean genes were decreased (with a FC ≥10) in *F*. *virguliforme-*infected roots as compared to that in the water control roots (Fig 1; Tables 1 and 2; S2 Dataset). We investigated the possible function of these infection-repressed genes for (i) molecular functions, (ii) biological processes and (iii) cellular locations through gene ontology (GO) analyses and results are presented in Figs 5–7.

GO analyses for molecular functions revealed that a large proportion of the down-regulated soybean genes do not show any identity to previously characterized genes and thus could be novel genes. For example, 40 genes (78%) with reduced transcript levels during ETP and 19 genes (78%) during LTP did not show homology to any functionally characterized genes (Fig 5). Infection-suppressed soybean genes encode proteins, most of which possess hydrolase and transferase activities at ETP (10% and 4% respectively) and lipid binding and transporter activities at LTP (11% for each category). Some of the repressed genes encode transferases, catalytic proteins, and transcription factors (Fig 5).

Reclassification of the down-regulated genes through GO analyses for biological processes again revealed that majority of the genes (83% in ETP and 96% in LTP) did not show identify to any genes with known biological processes. Down-regulated soybean genes with known biological processes during ETP include four (8%) genes for primary metabolic processes (lipid and carbohydrate metabolism), three (6%) for signal transduction, two (4%) for growth and cell death (Fig 6).

To shed light on the possible function of the down-regulated genes, we conducted GO analyses for putative sub-cellular location. Surprisingly, a significant proportion (15%) of the down-regulated genes encode plasma membrane proteins especially during ETP. These proteins may be involved in regulating signaling process during infection (Fig 7). Again, a large number of the genes (20 [39%] genes during and 14 [64%] in LTP) were found not to show similarity to any known functionally characterized genes.

Of the 51 genes repressed in soybean roots during ETP, only 15 with FC ≥15 continued to be suppressed during LTP following infection (Fig 1C; Tables 1 and 2, S2 Dataset). These genes include *Glyma*.*02g054200* encoding a SAM (salicylic acid methyltransferase) dependent carboxyl methyltransferase, *Glyma*.*15g082200* encoding a cysteine proteinase Cathepsin F, *Glyma*.*13g282200* and *Glyma*.*13g282400* encoding wound-induced proteins, *Glyma*.*16g178000* encoding a lipid transfer protein, and *Glyma*.*06g294400* and *Glyma*.*12g111500* encoding ankyrin-repeat containing proteins.

### Validation of transcriptomic data for a few selected genes repressed during SDS infection

To validate down-regulation of a few selected genes during infection, we performed a semi-quantitative reverse transcriptase polymerase chain reaction (RT-PCR) for one member from each of the four selected classes of down-regulated genes (Table 1). They are: (i) *Glyma*.*01g171600* encoding an uncharacterized salicylate o-methyltransferase (SAM)-like protein, we termed *GmSAM1*, most likely involved in the conversion of salicylic acid to the volatile methyl salicylate, a plant defense signal [56] for defending attack of necrotrophic pathogens including *F*. *virguliforme*; (ii) *Glyma12g12470* encoding an uncharacterized ankyrin repeat-containing protein, we termed it as *GmARP1;* (iii) *Glyma*.*10g185900* encodes a sieve element-occlusion protein (SEO)-like protein (http://www.uniprot.org; http://www.phytozome.net); and (iv) *Glyma*.*10g094800* encoding an uncharacterized, putative transmembrane protein [46](http://www.phytozome.net).

**Fig 8:**
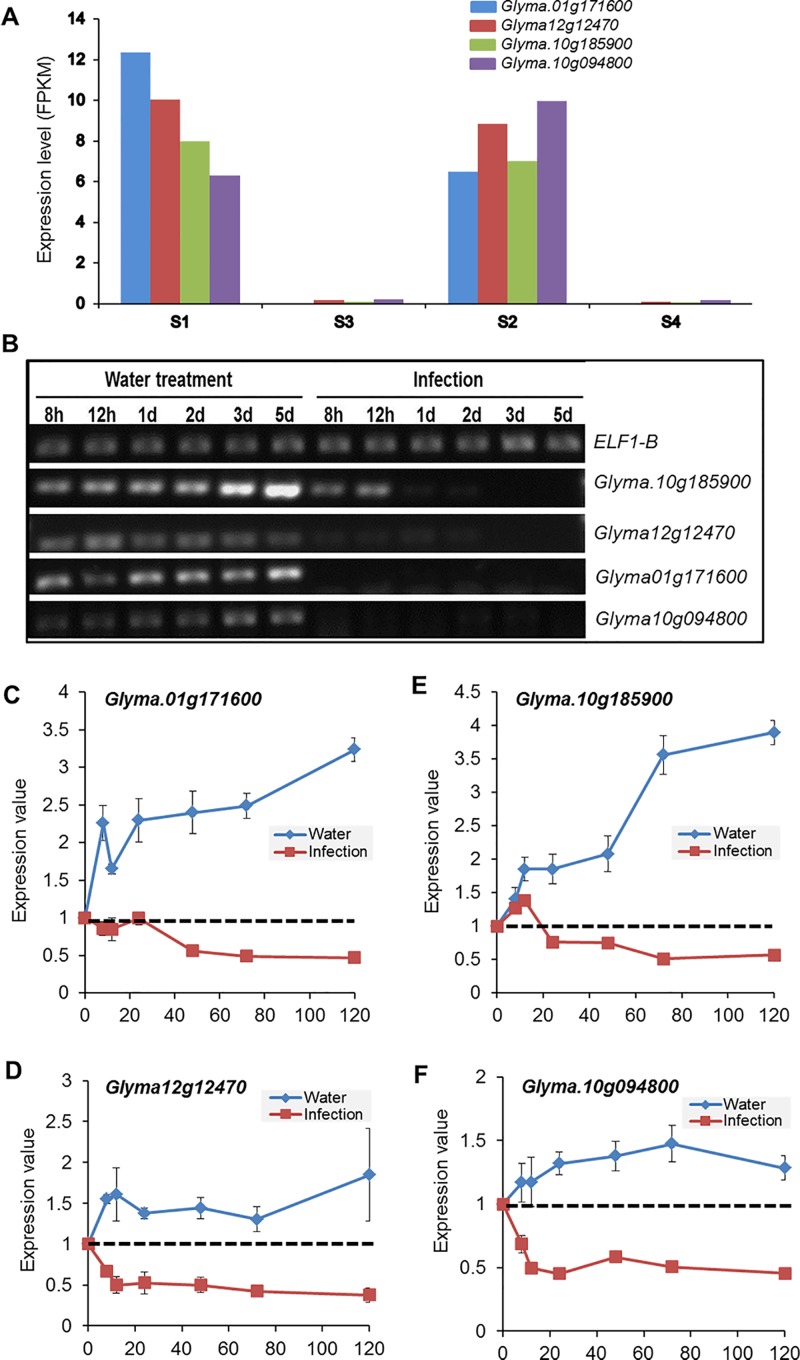
Reduced expression levels of soybean genes following *F*. *virguliforme* infection as compared to the water control. A. Expression levels of four selected soybean genes following water treatment at early (S1: 3 and 5 d) and late time (S2: 10 and 24 d) periods and *F*. *virguliforme* infection at early (S3: 3 and 5 d) and late time (S4: 10 and 24 d) periods. B. RT-PCR analyses of the selected soybean genes. RT-PCR products of each of the four selected soybean amplified from RNAs of root tissues harvested 8 and 12 h, and 1, 2, 3, 5 days following (i) water treatment or (ii) infection with the *F*. *virguliforme* Mont-1. The results presented here are from one of three independent experiments showing similar results. *Glyma12g12470* is from the Glyma.Wm82.a1.v1.1 version of the soybean genome sequence. Other three genes are from the recent version of the soybean genome sequence (Glyma.Wm82.a2v1). *Elf1b*, elongation factor 1-β encoded by *Glyma02g44460*. C–F. Quantified expression levels of four selected genes. Gel pictures of the three biological replications are presented in S8 Fig.

RNA samples of soybean roots harvested 8 and 12 h and 1, 2, 3 and 5 d post inoculation with *F*. *virguliforme* or treated with water were considered for RT-PCR. The results of the RT-PCR analysis confirmed the observation of the RNA seq experiment for the four selected genes of interest (Fig 8). RT-PCR data also suggested that the down-regulation of three of the four selected genes, *Glyma*.*01g171600*, *Glyma*.*10g094800*, and *Glyma12g12470*, is very rapid with little or no detectible transcript levels observed 8 h post inoculation.

### Alteration in expression of a *F*. *virguliforme*-repressed gene *GmARP1* showed foliar SDS resistance

We hypothesized that *F*. *virguliforme* down-regulates transcription of some of the defense-related genes to cause susceptibility in soybean. To test this hypothesis, we induced the expression of *GmARP1* (*Glyma12g12470*) in transgenic soybean plants following *F*. *virguliforme* infection. *Glyma*.*12g111500* and *Glyma*.*06g294400* are two highly similar genes that encode ankyrin repeats containing proteins with 76% identity (S4 and S5 Figs), but with no identity to any functionally characterized ankyrin repeat-containing genes including *GmNPR1* [57]. GmARP1 protein contains 218 residues and slightly larger than all known ankyrin repeat-containing proteins including ANK superfamily with 71–199 residues; ANK_2 with 81–171; ANK_4 with 145–199 residues.

We used three *F*. *virguliforme*-infection inducible promoters to induce the expression of *GmARP1* during *F*. *virguliforme* infection. Three fusion genes were generated by ligating *GmARP1* individually to these three promoters (S1A Fig) and used to transform soybean cultivar ‘Williams 82’. R_0_ plants were grown in a greenhouse and analyzed for integration of *GmARP1* transgenes by genomic PCR (S1B Fig).

**Fig 9:**
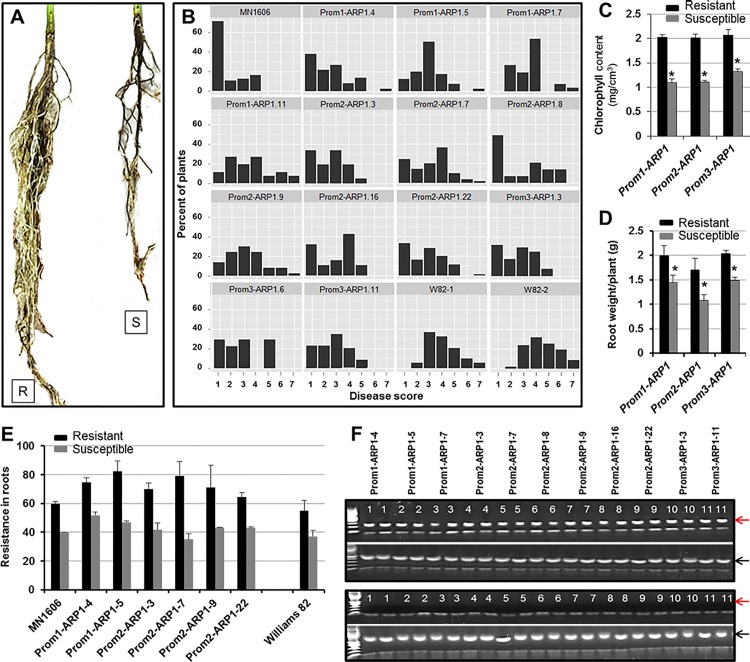
Expression of *GmARP1* enhances SDS resistance in transgenic soybean plants. R_1_ plants were tested for resistance to *F*. *virguliforme* under growth chamber conditions. A. Root phenotype of a resistant (R) and a susceptible (S) R_1_ progeny of a transformant, Prom2-ARP1-7, carrying the *Prom2-GmARP1* fusion gene. B. Enhanced foliar SDS resistance among R_1_ progenies. W82, the SDS susceptible line Williams 82; MN1606, the SDS resistant line. C. Chlorophyll content per individual R_1_ progeny carrying *GmARP1* of three independent transformants. ‘Resistant’ and ‘Susceptible’ classes are defined as in (A). D. Average root weight of R_1_ progeny of three independent transformants. ‘Resistant’ and ‘Susceptible’ classes are defined as in A. E. Enhanced root resistance among R_1_ progenies. Extent of root resistance to the pathogen was expressed in percent; e.g., 100%, healthy roots with no obvious blackening caused by necrosis and rotting due to infection of *F*. *virguliforme*. F. Expression of *GmARP1* transgenes. Two random SDS resistant and susceptible R_1_ progenies from each R_0_ line were analyzed. Top panel, resistant plants (two representatives from each line). Bottom panel, susceptible plants (two representatives from each line). Red arrow, *GmARP1*; black arrow, *ELF1b* internal control. *, significantly different at *p<0*.*01*. Results are means ±SE of three independent experiments.

R_1_ progenies of individual transformants (R_0_) carrying any of the three fusion *GmARP1* genes were inoculated with *F*. *virguliforme* Mont-1 isolate in a growth chamber (Fig 9A–9E). Approximately 30 to 50% of the R_1_ plants showed no symptoms to slight yellowing with disease scores 1 and 2. The rest showed severe disease symptoms with interveinal to severe chlorosis and necrosis with disease scores 3 to 7 (Fig 9B). On the contrary, susceptible R_1_ plants presumably lacking a functional transgene exhibited typical SDS foliar symptoms from the second week of infection and were severely diseased by the end of the fourth week following inoculation, with severe chlorosis and necrosis (disease scores 3 to 7), and reduced chlorophyll content as compared to the resistant R_1_ progeny plants (Fig 9C). Moreover, resistant transgenic plants showed increased root weight as compared to the susceptible R_1_ progenies implying root resistance of those resistant R_1_ progenies to the pathogen (Fig 9D) and is supported by the levels of root resistance observed among the R_1_ progenies (Fig 9E). Molecular characterization of the infected R_1_ plants indicated that expression of foliar and root SDS resistance among the R_1_ progenies of multiple transformants was associated with the expression of *GmARP1* transgenes (Fig 9F). No transcript of the endogenous *GmARP1* gene was detected during *F*. *virguliforme* infection.

**Fig 10:**
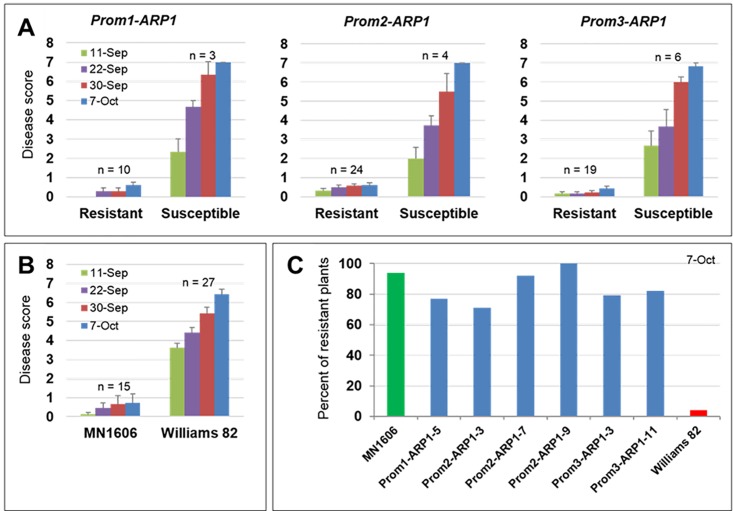
Expression of *GmARP1* enhances SDS resistance in transgenic soybean plants. R_1_ plants were tested for resistance to *F*. *virguliforme* under field conditions. A. Average disease scores gathered at four scoring dates for R_1_ progenies of each transgenic line carrying *GmARP1* under the control of Prom1, Prom2, or Prom3 promoters. n, number of R_1_ plants. B. Average disease scores of Williams 82 (susceptible control) and MN1606 (resistant control). n, number of plants and results in A and B are means ±SE of n plants. C. Percentage of R_1_ plants resistant to *F*. *virguliforme*.

We evaluated whether *GmARP1* transgenes could provide enhanced SDS resistance in transgenic plants under field conditions. We observed that 70 to 100% of the transgenic R_1_ plants descended from six independent transgenic R_0_ plants showed enhanced SDS resistance under field conditions and did not develop SDS symptoms. Non-trangenic Williams 82 control plants developed severe SDS symptoms including chlorosis and necrosis of leaves that caused total defoliation of plants before maturity stage (Fig 10A and 10C; S6 Fig). The transgene copy number assay conducted using qPCR revealed that all six transgenic lines contain at least one copy of the transgene, sufficient to enhance resistance against *F*. *virguliforme* (Table 3; S4 Dataset). The line Prom2-ARP1-9 carrying single copy *Prom2-ARP1* transgene showed 100% resistant R_1_ progenies; whereas, Prom2-ARP1-9 line carrying on the average 4 copies of the same *Prom2-ARP1* transgene showed the lowest number SDS resistant plants (70%).

**Table 3:** Average *GmARP1* transgene copy numbers among the transgenic lines.

Genotype	Transgene copy number	Number of plants	Average copy number (± SE)
	1	7	
Prom1-ARP1-5	2	4	2 (0.06)
	3	4	
	1	3	
	3	2	
Prom2-ARP1-3	4	3	4 (0.18)
	5	1	
	6	1	
	7	2	
	2	7	
Prom2-ARP1-7	3	1	3 (0.14)
	4	2	
	5	2	
	1	9	
Prom2-ARP1-9	2	2	1 (0.05)
	3	1	
Prom3-ARP1-3	2	10	2 (0.02)
	3	2	
	1	7	
Prom3-ARP1-11	2	4	2 (0.06)
	3	1	

## Discussion

Study of the steady state transcriptomes using next-generation sequencing is a powerful method for revealing candidate genes that may play roles in host-pathogen interactions. Alterations of the expression of defense-related genes have been reported in plants that are infected by fungi, particularly by *Fusaria* species [18,58]. Transcriptomic studies have revealed that more host genes are induced and fewer repressed following infection with fungal pathogens [19,21,58,59]. The majority of the induced genes are defense related, and it has been shown that overexpression of defense genes induced during infection in transgenic plants could enhance resistance to pathogens [32,60].

Here we report GO analyses of differentially expressed soybean genes identified by comparing transcriptomes of soybean roots following *F*. *virguliforme* infection with those of water treated soybean roots. A large number of the genes induced in soybean roots infected with *F*. *virguliforme* encode cell wall and plasma membrane proteins (Fig 4; S1 Dataset) and are presumably involved in generating protective barriers against the invading pathogens. A majority of proteins encoded by these genes have binding activities. It is known that proteins and enzymes that bind other proteins and metabolites are implicated in various metabolic functions. For instance, DNA binding proteins have been shown to recognize specific promoter sequences of the target defense genes for regulating defense mechanisms [61,62].

A set of infection-induced genes are involved in carbohydrate metabolism. Besides being primary metabolites in plant cells, sugars are also known as signals for plant induced responses to pathogen attacks [63,64]. Many of the genes induced in roots following *F*. *virguliforme* infection encode enzymes with catalytic or kinase activities. Some of the kinases are receptor kinases for receiving and transmitting signals [65] and are induced in roots during fungal attack [33,54]. Our data suggest that defense-related transcripts are induced following infection presumably to defend soybean against the *F*. *virguliforme* attack.

In the Arabidopsis-*F*. *oxysporum* interaction a large number of genes were repressed as opposed to induction as observed in most plant-pathogen interactions [66]. Despite a large number of soybean genes were up-regulated upon *F*. *virguliforme* infection, in our analysis of the soybean-*F*. *virguliforme* interaction revealed that 70 soybean genes were suppressed following infection. Of these, only four genes were down-regulated in both ETP and LTP following *F*. *virguliforme* infection (Fig 1C; S2 Dataset).

Among the genes down-regulated following *F*. *virguliforme* infection, seven soybean genes encode glycosyl hydrolases (Table 1). Of these three genes *Glyma*.*03g024400*, *Glyma*.*03g024300*, and *Glyma*.*03g024200* encoding the uncharacterized glycosyl hydrolase family 18 were highly expressed during ETP of the water treated root tissues (RPKM >1,102). The expression of these genes was strongly suppressed upon *F*. *virguliforme* infection (FC >65) (Table 1). Members of the glycosyl hydrolase 18 with high identity to some pathogen related (*PR*) genes are not only implicated in metabolic processes of cell wall, but also considered to have defense and signaling functions [67–70]. The glycosyl hydrolases encoded by *Glyma*.*03g024400*, *Glyma*.*03g024300*, and *Glyma*.*03g024200* are 100% identical and contain a GH18_chitinase-like domain; therefore most likely they function in plant defense against pathogens with chitin molecules [71,72]. Four additional soybean genes, *Glyma03g02810*, *Glyma*.*03g025000 Glyma*.*03g024800*, and *Glyma*.*08g285400* encoding glycosyl hydrolases with moderate expression levels (FC from 6 to 56), were also repressed during ETP following *F*. *virguliforme* infection.

Transcript levels of two genes *Glyma*.*09g022800* and *Glyma*.*09g023000* encoding uncharacterized peroxidases were strongly down-regulated following *F*. *virguliforme* infection (Table 1). The two proteins are 64% identical and contain a secretory peroxidase domain. Some peroxidases genes are also shown to be *PR* genes [73] and involved in plant defense against pathogens.

In our transcriptomic study, we included transcripts of roots of seedlings 3 and 5 days following *F*. *virguliforme* infection or water treatment for ETP. In the confirmatory RT-PCR experiment, we included roots of seedlings 8, 12 and 24 hours in addition to 3 and 5 days following infection or water treatment. Transcripts of all four genes included in the RT-PCR study were not detectable after 12 h following *F*. *virguliforme* infection (Fig 8; Table 1). Of these four genes, *Glyma*.*10g185900* (Fig 6A and 6B; Table 1; S2 Dataset) encodes a sieve element-occlusion protein (SEO), which is a structural protein implicated to play a role in plant defense [74,75]. Our data suggest that somehow *F*. *virguliforme* suppressed the expression of this gene to avoid any barrier arisen from this protein in the roots.

More than half of plant genes encode proteins of unknown functions [76]. We observed down-regulation of many genes encoding proteins of unknown functions during both ETP and LTP (FC > 10; Tables 1 and 2). During LTP, four genes have RPKM values in water treated roots over 1,000 (1,375 to 32,802) and are strongly repressed (FC ≥ 20) in *F*. *virguliforme* infected roots. The most highly expressed, *Glyma17g03850* (RPKM in water treated roots 32,802 and repressed after infection to FC = 24.0), is an orphan gene of *Glycine max* [77] (http://www.greenphyl.org/) with no annotated functional domain. This gene is only co-expressed with *Glyma*.*10G216000* (correlation 0.87, www.phytozome.com) that encodes a gibberellin-regulated protein 2 involved in gibberellic acid mediated signaling pathway [78]. Similarly, infection-suppressed gene *Glyma*.*10g232100* (RPKM 2,130 in water treated roots vs. FC 27 following infection) encoding an unknown protein also co-expressed with the gibberellin-regulated protein 2. Finally, *Glyma*.*17g039400* (RPKM 2,206 in water treated roots vs. FC 24 following infection). It is highly co-expressed with a fasciclin-like arabinogalactan protein gene (correlation 0.88, www.phytozome.com) implicated in the cell-wall composition [79]. Thus, these genes with unknown functions could be involved in plant immunity. Future studies on these four genes might yield new insights about the soybean-*F*. *virguliforme* interaction.

Aknyrin repeat-containing proteins widely exist in plants [80]. We identified five *GmARP1* homologs in *G*. *soja* and *Phaseolus vulgaris* exhibiting ≥ 62% identities among them, specifically in the ankyrin domain (S7 Fig). Two to more than 20 ankyrin-repeats involved in protein-protein interactions could be present in ankyrin-repeat containing proteins [81–83]. Ankyrin-repeats are involved in many metabolic processes including defense against pathogens and signaling [81,84–86]. The ankyrin repeat-containing ACD6 is implicated in salicylic acid signaling in Arabidopsis [87]. The ankyrin repeat-containing protein CaKR1 in *Capsicum annuum* is responsive to both abiotic and biotic stresses [88]. Ankyrin repeat-containing protein NPR1 modulates plant immunity cooperatively with transcription factors [89]. In Arabidopsis, BDA1 containing ankyrin repeats has been shown to regulate immunity [90]. Similarly, Mou and colleagues [60] reported that overexpression of the aknyrin repeat-containing protein gene *OsPIANK1* enhanced immunity against *Magnaporthe oryzae* in transgenic rice. *GmARP1* (*Glyma12g12470)* has been shown to have a protein binding function and putatively localized to plasma membrane [80]. Here we have shown that the steady state transcript level of this gene is not detectable immediately after *F*. *virguliforme* infection (Fig 8). Expression of this gene using infection-inducible promoters in transgenic plants enhanced resistance to the SDS pathogen, indicating that like other known aknyrin repeat-containing protein genes, *GmARP1* is involved in soybean defense against this fungal pathogen.

## Conclusions

Our transcriptomic study revealed many genes that are induced, whereas expression of only a few genes including a previously uncharacterized *GmARP1* gene is strongly suppressed by *F*. *virguliforme* infection. Many of the genes that are suppressed during infection might be defense-related and, are somehow suppressed by pathogens to establish the compatible interaction or to cause susceptibility. This hypothesis was tested by expressing *GmARP1* during *F*. *virguliforme* infection in transgenic soybean lines. We observed that expression of *GmARP1* led to induction of foliar SDS resistance. How *GmARP1* regulates immunity against SDS pathogen is yet to be determined. It is also unknown how the gene is down-regulated by *F*. *virguliforme* infection. Our transgenic study suggests that altered expression of pathogen-repressed host genes could be a suitable strategy in engineering disease resistance in crop plants.

## Supporting Information

S1 Fig Generation of *GmARP1* transgenes and R_0_ transgenic soybean plants. (DOCX)

S2 Fig *GmARP1* Sequences used in the transgenic study. (DOCX)

S3 Fig Development of a binary construct for transformation of soybean. (DOCX)

S4 Fig Screenshots of the *GmARP1* gene in the new and the old versions of genome sequence assemblies (www.soybase.org). (DOCX)

S5 Fig Sequence analysis of two ankyrin-repeat containing proteins downregulated following *F. virguliforme* infection. (DOCX)

S6 Fig Phenotype of transgenic soybean plants carrying *GmARP1* fusion genes in a field trial. (DOCX)

S7 Fig Sequence analysis of five ankyrin-repeat containing GmARP1 (*Glyma12g12470*) homologs. (DOCX)

S8 Fig RT-PCR analysis of three independent replicates for the four selected genes. (DOCX)

S1 Table List of primers used for PCR and RT-PCR (enzyme sites highlighted). (DOCX)

S2 Table Number of plants resistant to basta for *GmARP1* transgenic lines used in the field trial. (DOCX)

S3 Table Method of foliar SDS scoring in the field (www.siu.edu/~soybean). (DOCX)

S4 Table List of primers used for RT-PCR of five selected genes in the infected soybean roots. (DOCX)

S1 Dataset Genes up-regulated in soybean roots infected with *F. virguliforme* at early time period. (XLSX)

S2 Dataset Genes down-regulated at early time period in soybean roots infected by *F. virguliforme*. (XLSX)

S3 Dataset Genes up-regulated in soybean roots infected by *F. virguliforme* at late time period. (XLSX)

S4 Dataset Transgene copy number analysis by qPCR. (XLSX)

## References

[1] Wrather JA, Anderson TR, Arsyad DM, Gai J, Ploper LD, Porta-Puglia A, et al Soybean disease loss estimates for the top 10 soybean producing countries in 1994. Plant Dis. 1997; 81: 107–110. 10.1094/PDIS.1997.81.1.107 30870925

[2] Wrather JA, Koenning SR. Estimates of disease effects on soybean yield in United States 2003 to 2005. J Nematol. 2006; 38: 173–180. 19259444 PMC2586459

[3] Koenning SR, Wrather JA. Suppression of soybean yield potential in the continental United States by plant diseases from 2006 to2009. Plant Health Progress. 2010. doi: 101094/PHP-2010-1122-01-RS.

[4] Aoki T, O'Donnell K, Homma Y, Lattanzi A. Sudden-death syndrome of soybean is caused by two morphologically and phylogenetically distinct species within the *Fusarium solani* species complex- *F*. *virguliforme* in North America and *F*. *tucumaniae* in South America. Mycologia. 2003; 95: 660–684. 21148975

[5] Aoki T, O’Donnell K, Scandiani MM. Sudden death syndrome of soybean in South America is caused by four species of *Fusarium*: *Fusarium brasiliense* sp. nov., *F*. *cuneirostrum* sp. nov., *F*. *tucumaniae*, and *F*. *virguliforme*. Mycoscience. 2005; 46: 162–183.

[6] Hughes TJ, O’Donnell K, Sink S, Rooney AP, Scandiani MM, Luque A, et al Genetic architecture and evolution of the mating type locus in *fusaria* that cause soybean sudden death syndrome and bean root rot. Mycologia. 2014; 106: 686–697. 10.3852/13-318 24891421

[7] Njiti VN, Suttner RJ, Gray LE, Gibson PP, Lightfoot DA. Rate reducing resistance to *Fusarium solani* f. sp. *Phaseoli* underlies field resistance to soybean sudden death syndrome. Crop Sci. 1997; 37: 132–138.

[8] Gao X, Jackson K, Lambert K, Li S, Niblack T, Hartman G. Detection and quantification of *Fusarium solani* f.sp. *glycines* in soybean roots with real-time quantitative polymerase chain reaction. Plant Dis. 2004; 88: 1372–1380. 10.1094/PDIS.2004.88.12.1372 30795200

[9] Jin H, Hartman GL, Nickel CD, Widholm JM. Phytotoxicity of culture filtrates from *Fusarium solani*, the causal agent of sudden death syndrome of soybean. Plant dis. 1996; 80: 922–927.

[10] Brar HK, Swaminathan S, Bhattacharyya MK. The *Fusarium virguliforme* toxin FvTox1 causes foliar sudden death syndrome-like symptoms in soybean. Mol Plant-Microbe Interact. 2011; 24: 1179–1188. 10.1094/MPMI-12-10-0285 21635141

[11] Brar HK, Bhattacharyya MK. Expression of a single-chain variable-fragment antibody against a *Fusarium virguliforme* toxin peptide enhances tolerance to sudden death syndrome in transgenic soybean plants. Mol Plant-Microbe Interact. 2012; 25: 817–824. 10.1094/MPMI-12-11-0317 22397408

[12] Pudake RN, Swaminathan S, Sahu BB, Leandro LF, Bhattacharyya MK. Investigation of the *Fusarium virguliforme fvtox1* mutants revealed that the FvTox1 toxin is involved in foliar sudden death syndrome development in soybean. Current Genet. 2013; 59: 107–117. 10.1007/s00294-013-0392-z 23702608

[13] Chang HX, Domier LL, Radwan O, Yendrek CR, Hudson ME, Hartman GL. Identification of multiple phytotoxins produced by *Fusarium virguliforme* including a phytotoxic effector (FvNIS1) associated with sudden death syndrome foliar symptoms. Mol Plant-Microbe Interact. 2016; 29: 96–108. 10.1094/MPMI-09-15-0219-R 26646532

[14] Abeysekara NS, Bhattacharyya MK. Analyses of the xylem sap proteomes identified candidate *Fusarium virguliforme* proteinacious toxins. PLoS One. 2014; 9: e93667 10.1371/journal.pone.0093667 24845418 PMC4028188

[15] Zou J, Rodriguez-Zas S, Aldea M, Li M, Zhu J, Gonzalez DO, et al Expression profiling soybean response to *Pseudomonas syringae* reveals new defense-related genes and rapid HR-specific downregulation of photosynthesis. Mol Plant-Microbe Interact. 2005; 18: 1161–1174. 10.1094/MPMI-18-1161 16353551

[16] Zabala G, Zou J, Tuteja J, Gonzalez DO, Clough SJ, Vodkin LO. Transcriptome changes in the phenylpropanoid pathway of *Glycine max* in response to *Pseudomonas syringae* infection. BMC Plant Biol. 2006; 6: 26 10.1186/1471-2229-6-26 17083738 PMC1636052

[17] Yuan JZ, Zhu MX, Lightfoot DA, Iqbal MJ, Yang JY, Meksem K. In silico comparison of transcript abundances during *Arabidopsis thaliana* and *Glycine max* resistance to *Fusarium virguliforme*. BMC Genomics. 2008; 9 (Suppl 2): S6 10.1186/1471-2164-9-S2-S6 18831797 PMC2559896

[18] D'Ippólito S, Martin LM, Salcedo MF, Atencio HM, Casalongue CA, Godoy AV, Fiol DF. Transcriptome profiling of *Fusarium solani* f. sp. *eumartii*-infected potato tubers provides evidence of an inducible defense response. Physiol Mol Plant Path. 2010; 75: 3–12.

[19] Moy P, Qutob D, Chapman BP, Atkinson I, Gijzen M. Patterns of gene expression upon infection of soybean plants by *Phytophthora sojae*. Mol Plant-Microbe Interact. 2004; 17: 1051–1062. 10.1094/MPMI.2004.17.10.1051 15497398

[20] Iqbal MJ, Yaegashi S, Ahsan R, Shopinski KL, Lightfoot DA. Root response to *Fusarium solani* f. sp. *glycines*: temporal accumulation of transcripts in partially resistant and susceptible soybean. Theor Appl Genet. 2005; 110: 1429–1438. 10.1007/s00122-005-1969-9 15815926

[21] Radwan O, Liu Y, and Clough SJ. Transcriptional analysis of soybean root response to *Fusarium virguliforme*, the causal agent of sudden death syndrome. Mol Plant-Microbe Interact. 2011; 24: 958–972. 10.1094/MPMI-11-10-0271 21751852

[22] Roy K, Rupe JC. Sudden death syndrome of soybean. Plant Dis. 1997; 81: 259–266. 10.1094/PDIS.1997.81.10.1100 30861702

[23] Rupe JC. Frequency and pathogenicity of *Fusarium solani* recovered from soybeans with sudden death syndrome. Plant Dis. 1989; 73: 581–584.

[24] Anderson TR, Tenuta A. First report of *Fusarium solani* f. sp. *glycines* causing sudden-death syndrome of soybean in Canada. Plant Dis. 1998; 82: 448. 10.1094/PDIS.1998.82.4.448D 30856905

[25] Leandro LF, Tatalovic N, Luckew A. Soybean sudden death syndrome—Advances in knowledge and disease management. CAB Rev. 2012; 7: 1–14.

[26] Stephens PA, Nickell CD, Kolb FL. Genetic-analysis of resistance to *Fusarium solani* in soybean. Crop Sci. 1993; 33: 929–930.

[27] Njiti VN, Meksem K, Iqbal MJ, Johnson JE, Kassem M, Zobrist KF, et al Common loci underlie field resistance to soybean sudden death syndrome in Forrest, Pyramid, Essex, and Douglas. Theor Appl Genet. 2002; 104: 294–300. 10.1007/s001220100682 12582700

[28] Kassem MA, Ramos L, Leandro LF, Mbofung GYC, Hyten DL, Kantartzi SK, et al The ‘PI 438489B’ by ‘Hamilton’ SNP-based genetic linkage map of soybean [*Glycine max* (L.) Merr.] identified quantitative trait loci that underlie seedling SDS resistance. J Plant Gen Sci. 2012; 1: 18–30.

[29] Wen Z, Tan R, Yuan J, Bales C, Du W, Zhang S, et al Genome-wide association mapping of quantitative resistance to sudden death syndrome in soybean. BMC Genomics. 2014; 15: 809 10.1186/1471-2164-15-809 25249039 PMC4189206

[30] Swaminathan S, Abeysekara NS, Liu M, Cianzio SR, Bhattacharyya MK. Identification of quantitative trait loci underlying the sensitivity of soybean to the *Fusarium virguliforme* toxins that induce foliar soybean sudden death syndrome in soybean. Theor Appl Genet. 2016; 129: 495–506. 10.1007/s00122-015-2643-5 26678962

[31] Kesarwani M, Azam M, Natarajan K, Mehta A, Datta A. Oxalate decarboxylase from Collybia velutipes. Molecular cloning and its overexpression to confer resistance to fungal infection in transgenic tobacco and tomato. J Biol Chem. 2000; 275: 7230–7238. 10702293 10.1074/jbc.275.10.7230

[32] Cunha WG, Tinoco MLP, Pancoti HL, Ribeiro RE, Aragão FJL. High resistance to *Sclerotinia sclerotiorum* in transgenic soybean plants transformed to express an oxalate decarboxylase gene. Plant Path. 2010; 59: 654–660.

[33] Rey T, Nars A, Bonhomme M, Bottin A, Huguet S, Balzergue S, Jardinaud MF, Bono JJ, Cullimore J, Dumas B, Gough C, Jacquet C. NFP, a LysM protein controlling Nod factor perception, also intervenes in *Medicago truncatula* resistance to pathogens. New Phytologist. 2013; 198: 875–886. 10.1111/nph.12198 23432463

[34] Catanzariti AM, Lim GTT, Jones DA. The tomato *I-3* gene: a novel gene for resistance to *Fusarium* wilts disease. New Phytol. 2015; 207: 106–118. 10.1111/nph.13348 25740416

[35] Bhattacharyya MK, Ward EWB. Resistance, susceptibility and accumulation of glyceollin I-III in soybeans inoculated with *Phytophthora megasperma* f. sp. *glycinea*. Physiol Mol Plant Pathol. 1986; 29: 227–237.

[36] Ngaki MN, Louie GV, Philippe RN, Manning G, Pojer F, Bowman ME, et al Evolution of the chalcone-isomerase fold from fatty-acid binding to stereospecific catalysis. Nature. 2012; 485: 530–533. 10.1038/nature11009 22622584 PMC3880581

[37] Doyle JJ, Doyle JL. Isolation of plant DNA from fresh tissue. BRL Focus. 1990; 12: 13–15.

[38] Frame BR, Shou HX, Chikwamba RK, Zhang ZY, Xiang CB, Fonger TM, et al *Agrobacterium tumefaciens*-mediated transformation of maize embryos using a standard binary vector system. Plant Physiol. 2002; 129: 13–22. 10.1104/pp.000653 12011333 PMC1540222

[39] Luckew AS, Cianzio SR, Leandro LF. Screening method for distinguishing soybean resistance to *Fusarium virguliforme* in resistant by resistant crosses. Crop Sci. 2012; 52: 2215–2223.

[40] Hartman GL, Huang YH, Nelson RL, Noel GR. Germplasm evaluation of *Glycine max* for resistance to *Fusarium solani*, the causal organism of sudden death syndrome. Plant Dis. 1997; 81: 515–518. 10.1094/PDIS.1997.81.5.515 30861933

[41] Mueller DS, Nelson RL, Hartman GL, Pedersen WL. Response of commercially developed soybean cultivars and the ancestral soybean lines to *Fusarium solani* f. sp. *glycines*. Plant Dis. 2003; 87: 827–831. 10.1094/PDIS.2003.87.7.827 30812894

[42] Li S, Hartman GL, Chen Y. Evaluation of aggressiveness of *Fusarium virguliforme* isolates that cause soybean sudden death syndrome. J Plant Path. 2009; 91: 77–86.

[43] Arnon DI. Copper enzymes in isolated chloroplasts and polyphenol oxidase in *Beta vulgaris*. Plant Physiol. 1949; 24: 1–15. 16654194 10.1104/pp.24.1.1 PMC437905

[44] Langmead B, Trapnell C, Pop M, Salzberg S. Ultrafast and memory-efficient alignment of short DNA sequences to the human genome. Genome Biol. 2009; 10:R25 10.1186/gb-2009-10-3-r25 19261174 PMC2690996

[45] Mortazavi A, Williams BA, McCue K, Schaeffer L, Wold B. Mapping and quantifying mammalian transcriptomes by RNA-Seq. Nat Meth. 2008; 5:621–628. 10.1038/nmeth.1226 18516045 PMC13303166

[46] Goodstein DM, Shu S, Howson R, Neupane R, Hayes RD, Fazo J, et al Phytozome: a comparative platform for green plant genomics. Nucleic Acids Res. 2012; 40: 1178–1186. 10.1093/nar/gkr944 PMC3245001 22110026

[47] Abramoff M, Magelhaes P, Ram S. 2004. Image Processing with ImageJ. Biophotonics International 2004; 11: 36–42.

[48] Schmutz J, Cannon SB, Schlueter J, Ma J, Mitros T, Nelson W, et al Genome sequence of the palaeopolyploid soybean. Nature. 2010; 465: 178–183. 10.1038/nature08670 20075913

[49] Valdés-López O, Thibivilliers S, Qiu J, Xu WW, Nguyen TH, Libault M, et al Identification of quantitative trait loci controlling gene expression during the innate immunity response of soybean. Plant Physiol. 2011; 157: 1975–1986. 10.1104/pp.111.183327 21963820 PMC3327182

[50] Grant D, Nelson RT, Cannon SB, Shoemaker RC. SoyBase, the USDA-ARS soybean genetics and genomics database. Nucleic Acids Res. 2010; 38: D843–D846. 10.1093/nar/gkp798 20008513 PMC2808871

[51] Mizutani M, Ohta D. Diversification of P450 genes during land plant evolution. Annual Rev Plant Biol. 2010; 61: 291–315. 20192745 10.1146/annurev-arplant-042809-112305

[52] Choi JJ, Alkharouf NW, Schneider KT, Matthews BF, Frederick RD. Expression patterns in soybean resistant to *Phakopsora pachyrhizi* reveal the importance of peroxidases and lipoxygenases. Funct Integr Genomics. 2008; 8: 341–359. 10.1007/s10142-008-0080-0 18414911

[53] Antolin-Llovera M, Ried MK, Binder A, Parniske M. Receptor kinase signaling pathways in plant-microbe interactions. Ann Rev Phytopath. 2012; 50: 451–473. 10.1146/annurev-phyto-081211-173002 22920561

[54] Uppalapati SR, Marek SM, Lee HK, Nakashima J, Tang Y, Sledge MK, et al Global gene expression profiling during *Medicago Truncatula*-*Phymatotrichopsis omnivora* interaction reveals a role for jasmonic acid, ethylene, and the flavonoid pathway in disease development. Mol Plant-Microbe Interact. 2009; 22: 7–17. 10.1094/MPMI-22-1-0007 19061398

[55] Kombrink A, Sanchez-Vallet A, Thomma BP. The role of chitin detection in plant-pathogen interactions. Microb Infect. 2011; 13: 1168–1176. 10.1016/j.micinf.2011.07.010 21856436

[56] Lin J, Mazarei M, Zhao N, Zhu JJ, Zhuang X, Li W, et al Overexpression of a soybean salicylic acid methyltransferase gene confers resistance to soybean cyst nematode. Plant Biotech J. 2013; 11: 1135–1145. 10.1111/pbi.12108 24034273

[57] Sandhu DS, Tasma IM, Frasch R, Bhattacharyya MK. Systemic acquired resistance in soybean is regulated by two proteins, orthologous to Arabidopsis NPR1. BMC Plant Biol. 2009; 9: 105 10.1186/1471-2229-9-105 19656407 PMC2738679

[58] Zhu QH, Stephen S, Kazan K, Jin G, Fan L, Taylor J, et al Characterization of the defense transcriptome responsive to *Fusarium oxysporum*-infection in Arabidopsis using RNA-seq. Gene. 2013; 512: 259–66. 10.1016/j.gene.2012.10.036 23107761

[59] Lanubile A, Ferrarini A, Maschietto V, Delledonne M, Marocco A, Bellin D. Functional genomic analysis of constitutive and inducible defense responses to *Fusarium verticillioides* infection in maize genotypes with contrasting ear rot resistance. BMC Genomics. 2014; 15: 710 10.1186/1471-2164-15-710 25155950 PMC4153945

[60] Mou S, Liu Z, Guan D, Qiu A, Lai Y, He S. Functional analysis and expressional characterization of rice ankyrin repeat-containing protein, OsPIANK1, in basal defense against *Magnaporthe oryzae* attack. PLoS ONE. 2013; 8: e59699 10.1371/journal.pone.0059699 23555750 PMC3608567

[61] Chen C, Chen Z. Isolation and characterization of two pathogen- and salicylic acid-induced genes encoding WRKY DNA-binding proteins from tobacco. Plant Mol Biol. 2000; 42: 387–396. 10794538 10.1023/a:1006399311615

[62] Yu D, Chen C, Chen Z. Evidence for an important role of WRKY DNA binding proteins in the regulation of *NPR1* gene expression. Plant Cell. 2001; 13: 1527–1540. 10.1105/TPC.010115 11449049 PMC139550

[63] Trewavas A. Plant cell signal transduction: the emerging phenotype. Plant cell. 2002; 14: S3–S4. 10.1105/tpc.141360 12045265 PMC209458

[64] Vorwerk S, Somerville S, Somerville C. The role of plant cell wall polysaccharide composition in disease resistance. Trends Plant Sci. 2004; 9: 203–209. 10.1016/j.tplants.2004.02.005 15063871

[65] Zipfel C. Plant pattern-recognition receptors. Trends Immunol. 2014; 35: 345–351. 10.1016/j.it.2014.05.004 24946686

[66] Chen YC, Wong CL, Muzzi F, Vlaardingerbroek I, Kidd BN, Schenk PM. Root defense analysis against *Fusarium oxysporum* reveals new regulators to confer resistance. Scientific Reports. 2014; 4: 5584 10.1038/srep05584 24998294 PMC4083284

[67] Bowles DJ. Defense-related proteins in higher plants. Ann Rev Biochem. 1990; 59: 873–907. 10.1146/annurev.bi.59.070190.004301 2197993

[68] Datta SK, Muthukrishnan S. Pathogenesis-related proteins in plants 1^st^ ed. Boca Raton, Florida: CRC Press LLC; 1999.

[69] van der Holst PPG, Schlaman HRM, Spaink HP. Proteins involved in the production and perception of oligosaccharides in relation to plant and animal development. Curr Opin Struct Biol. 2001; 11: 608–616. 11785763 10.1016/s0959-440x(00)00255-4

[70] Minic Z. Physiological roles of plant glycoside hydrolases. Planta. 2008; 227: 723–740. 10.1007/s00425-007-0668-y 18046575

[71] Neuhaus JM, Fritig B, Linthorst HJM, Meins FJ. A revised nomenclature for chitinase genes. Plant Mol Biol Rep. 1996; 141: 102–104.

[72] Passarinho PA, de Vries SC. Arabidopsis chitinases: a genomic survey. Arabidopsis Book. 2002; 1: e0023 10.1199/tab.0023 22303199 PMC3243303

[73] Van Loon LC, Rep M, Pieterse CMJ. Significance of inducible defence-related proteins in infected plants. Ann Rev Phytopath. 2006; 44: 135–162. 10.1146/annurev.phyto.44.070505.143425 16602946

[74] Noll G, Rüping B, Ernst A, Bucsenez M, Twyman R, Fischer R, Prüfer D. The Promoters of forisome genes *MtSEO2* and *MtSEO3* direct gene expression to immature sieve elements in *Medicago truncatula* and *Nicotiana tabacum*. Plant Mol Biol Rep. 2009; 27: 526–533.

[75] Ruping B, Ernst A, Jekat S, Nordzieke S, Reineke A, Muller B, et al Molecular and phylogenetic characterization of the sieve element occlusion gene family in fabaceae and non-fabaceae plants. BMC Plant Biol. 2010; 10: 219 10.1186/1471-2229-10-219 20932300 PMC3017817

[76] Niehaus TD, Thamm AMK, de Crécy-Lagard V, Hanson AD. Proteins of unknown biochemical function: a persistent problem and a roadmap to help overcome it. Plant Physiol. 2015; 169: 1436–1442. 10.1104/pp.15.00959 26269542 PMC4634069

[77] Rouard M, Guignon V, Aluome C, Laporte MA, Droc G, Walde C, et al GreenPhylDB v2.0: comparative and functional genomics in plants. Nucl Acids Res. 2010; 39: D1095–D1102. 10.1093/nar/gkq811 20864446 PMC3013755

[78] Takasaki H, Mahmood T, Matsuoka M, Matsumoto H, Komatsu S. Identification and characterization of a gibberellin-regulated protein, which is ASR5, in the basal region of rice leaf sheaths. Mol Genet Genomics. 2008; 279: 359–370. 10.1007/s00438-007-0317-y 18210155

[79] Wang HH, Jiang CM, Wang CT, Yang Y, Gao XY, Zhang HX. Antisense expression of the fasciclin-like arabinogalactan protein PtFLA6 gene in *Populus* inhibits expression of its homologous genes and alters stem biomechanics and cell wall composition in transgenic trees. J Exp Bot. 2015; 66: 1291–1302. 10.1093/jxb/eru479 25428999 PMC4339592

[80] Becerra C, Jahrmann T, Puigdomenech P, Vicient CM. Ankyrin repeat-containing proteins in Arabidopsis: characterization of a novel and abundant group of genes coding ankyrin-transmembrane proteins. Gene. 2004; 340: 111–121. 10.1016/j.gene.2004.06.006 15556299

[81] Li HY, Chye ML. Arabidopsis Acyl-CoA-binding protein ACBP2 interacts with an ethylene-responsive element-binding protein, AtEBP, via its ankyrin repeats. Plant Mol Biol. 2004; 54: 233–243. 10.1023/B:PLAN.0000028790.75090.ab 15159625

[82] Böttner S, Iven T, Carsjens CS, Dröge-Laser W. Nuclear accumulation of the ankyrin repeat protein ANK1 enhances the auxin-mediated transcription accomplished by the bZIP transcription factors BZI-1 and BZI-2. Plant J. 2009; 58: 914–926. 10.1111/j.1365-313X.2009.03829.x 19220790

[83] Shen G, Kuppu S, Venkataramani S, Wang J, Yan J, Qiu X, et al Ankyrin repeat-containing protein 2A is an essential molecular chaperone for peroxisomal membrane-bound ascorbate peroxidase3 in Arabidopsis. Plant Cell. 2010; 22: 811–831. doi: 10.1105/tpc.109.065979 20215589 PMC2861468

[84] Yan J, Wang J, Zhang H. An ankyrin repeat-containing protein plays a role in both disease resistance and antioxidation metabolism. Plant J. 2002; 29: 193–202. 11862948 10.1046/j.0960-7412.2001.01205.x

[85] Zhang H, Li X, Zhang Y, Kuppu S, Shen G. Is AKR2A an essential molecular chaperone for a class of membrane-bound proteins in plants? Plant Sign Behav. 2010; 5: 1520–1522. 10.4161/psb.5.11.13714 PMC3115272 21057222

[86] Carvalho SD, Saraiva R, Maia TM, Abreu IA, Duque P. XBAT35, a novel Arabidopsis RING E3 ligase exhibiting dual targeting of its splice isoforms, is involved in ethylene-mediated regulation of apical hook curvature. Mol Plant. 2012; 5: 1295–1309. 10.1093/mp/sss048 22628544

[87] Lu H, Rate DN, Song JT, Greenberg JT. ACD6, a novel ankyrin protein, is a regulator and an effector of salicylic acid signaling in the Arabidopsis defense response. Plant Cell. 2003; 15: 2408–2420. 10.1105/tpc.015412 14507999 PMC197305

[88] Seong ES, Choi D, Cho HS, Lim CK, Cho HJ, Wang MY. Characterization of a stress-responsive ankyrin repeat-containing zinc finger protein of *Capsicum annuum* (CaKR1). J Biochem Mol Biol. 2007; 40: 952–958. 18047791 10.5483/bmbrep.2007.40.6.952

[89] Cao H, Glazebrook J, Clarke JD, Volko S, Dong X. The Arabidopsis *NPR1* gene that controls systemic acquired resistance encodes a novel protein containing ankyrin repeats. Cell. 1997; 88: 57–63. 9019406 10.1016/s0092-8674(00)81858-9

[90] Yang Y, Zhang Y, Ding P, Johnson K, Li X, Zhang Y. The ankyrin-repeat transmembrane protein BDA1 functions downstream of the receptor-like protein SNC2 to regulate plant immunity. Plant Physiol. 2012; 159: 1857–1865. 10.1104/pp.112.197152 22740615 PMC3425218

