# Supplementary material for: Tanscriptomic Study of the Soybean-Fusarium virguliforme Interaction Revealed a Novel Ankyrin-Repeat Containing Defense Gene, Expression of Whose during Infection Led to Enhanced Resistance to the Fungal Pathogen in Transgenic Soybean Plants
Source: PLoS One. 2016 Oct 19;11(10):e0163106. doi: 10.1371/journal.pone.0163106 (PMC5070833; doi:10.1371/journal.pone.0163106)
Supplement: S1 Fig — (DOCX) [file pone.0163106.s001.docx]

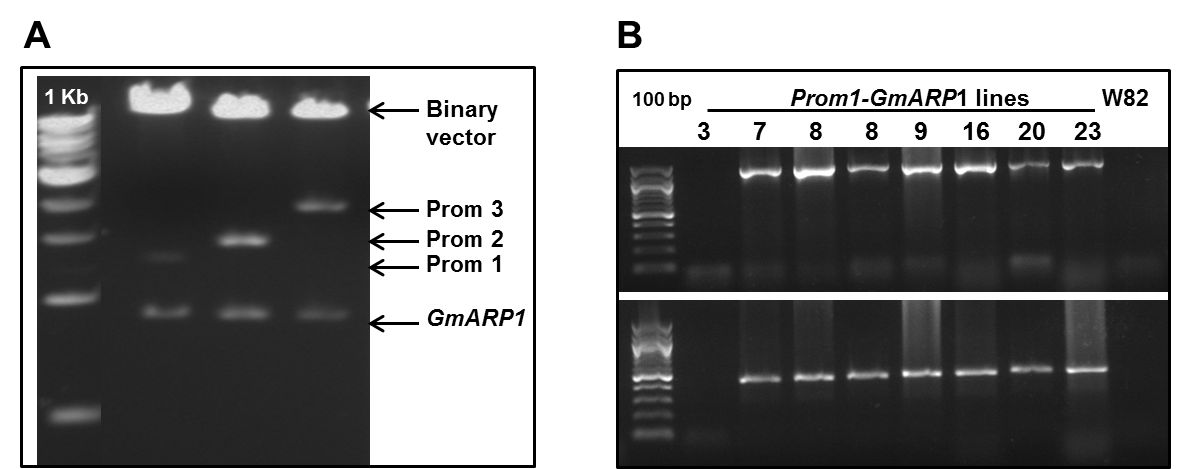


**S1 Fig. Generation of *GmARP1* transgenes and R_0_ transgenic soybean plants. (**a) Generation of three *GmARP1* transgenes*.* The *GmARP1* gene was fused to three promoters: Prom 1 (*Glyma18g47390*), Prom 2 (*Glyma10g31210*), and Prom 3 (*Glyma20g36300*). Binary plasmids carrying three fusion genes generated by fusing *GmARP1* to each ­of the three promoters were digested with *Xba*I and *Bst*XI. (b) Molecular characterization of soybean transformants carrying the *Prom2-GmARP1* transgene. (i) PCR amplification of *GmARP1* transgene using one *GmARP1*-specific and one vector-specific primer. (ii) PCR amplification of the *bar* gene. Lines 3, 7, through 23 are independent R_0_ plants.
