# Supplementary material for: Tanscriptomic Study of the Soybean-Fusarium virguliforme Interaction Revealed a Novel Ankyrin-Repeat Containing Defense Gene, Expression of Whose during Infection Led to Enhanced Resistance to the Fungal Pathogen in Transgenic Soybean Plants
Source: PLoS One. 2016 Oct 19;11(10):e0163106. doi: 10.1371/journal.pone.0163106 (PMC5070833; doi:10.1371/journal.pone.0163106)
Supplement: S2 Fig — (DOCX) [file pone.0163106.s002.docx]

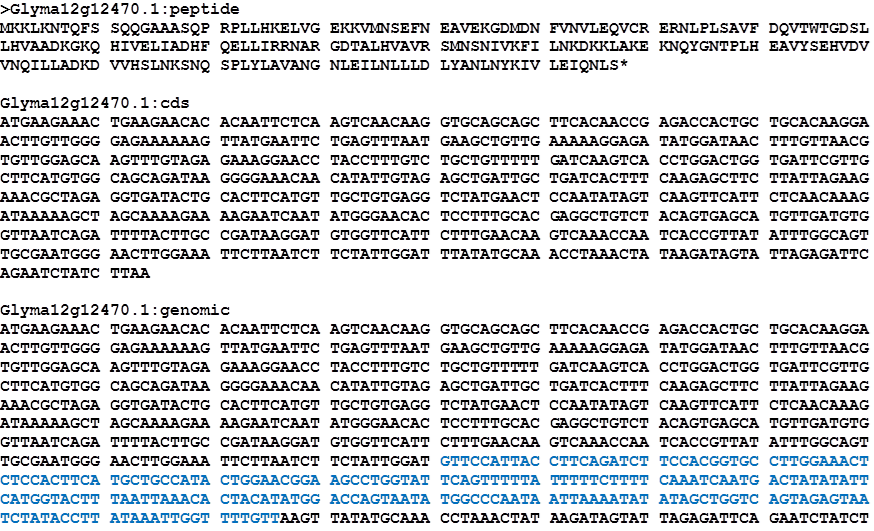


**S2 Fig. *GmARP1* Sequences used in the transgenic study.** Peptide, the deduced amino acid coding sequence of *Glyma12g12470;* cds, coding sequence of GmARP1; Genomic, *Glyma12g12470* sequence including two exons (black) and one intron (blue).
