# Supplementary material for: Tanscriptomic Study of the Soybean-Fusarium virguliforme Interaction Revealed a Novel Ankyrin-Repeat Containing Defense Gene, Expression of Whose during Infection Led to Enhanced Resistance to the Fungal Pathogen in Transgenic Soybean Plants
Source: PLoS One. 2016 Oct 19;11(10):e0163106. doi: 10.1371/journal.pone.0163106 (PMC5070833; doi:10.1371/journal.pone.0163106)
Supplement: S3 Fig — (DOCX) [file pone.0163106.s003.docx]

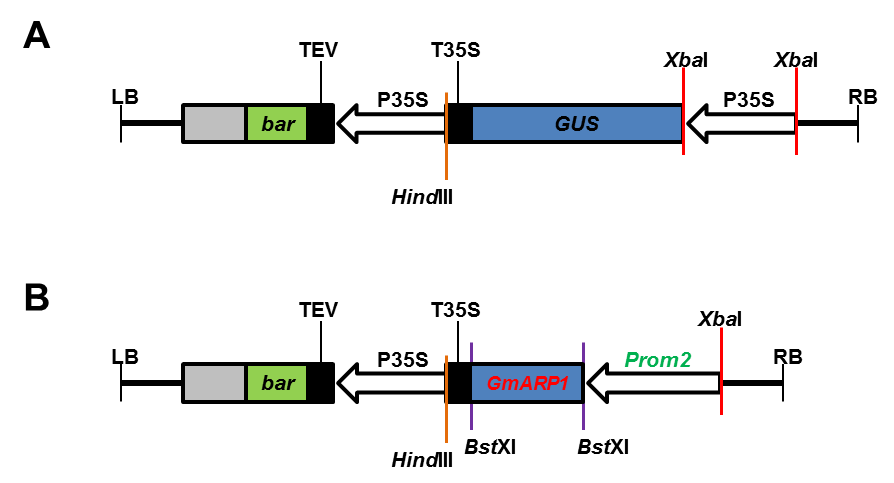


**S3 Fig. Development of a binary construct for transformation of soybean.** (A) T-DNA region of binary vector pTF102 (Frame *et al*., 2002**)**. (B) The binary vector pTF102 was used to create three *GmARP1* transgenes: *Prom1-GmARP1*, *Prom2-GmARP1*, and *Prom3-GmARP1* as follows. First, the CaMV 35S promoter was removed from pTF102 by digesting with *Xba*I and replaced it with any of the three new promoters. The restriction site for cloning *GmARP1* (*Bst*XI) was inserted at the 3’-end of the promoter primers. Next, we excised the *GUS* gene and CaMV 35S terminator (containing the PolyA signal) by digesting with *Bst*XI and the *Hind*III. The CaMV 35S terminator was reinserted with addition of the *Bst*XI at the 5’-end, and cloned in the *Bst*XI and *Hind*III sites. Finally, the created vectors were digested with *Bst*XI and the sequence of *GmARP1* including 84 nucleotides upstream of the ATG start codon and 45 nucleotides beyond the TAA stop codon. LB, Left border; RB, right border; P35S, CaMV 35S promoter; TEV, tobacco etch virus translational enhancer; T35S, CaMV 35S terminator.
