# Supplementary material for: Tanscriptomic Study of the Soybean-Fusarium virguliforme Interaction Revealed a Novel Ankyrin-Repeat Containing Defense Gene, Expression of Whose during Infection Led to Enhanced Resistance to the Fungal Pathogen in Transgenic Soybean Plants
Source: PLoS One. 2016 Oct 19;11(10):e0163106. doi: 10.1371/journal.pone.0163106 (PMC5070833; doi:10.1371/journal.pone.0163106)
Supplement: S4 Fig — (DOCX) [file pone.0163106.s004.docx]

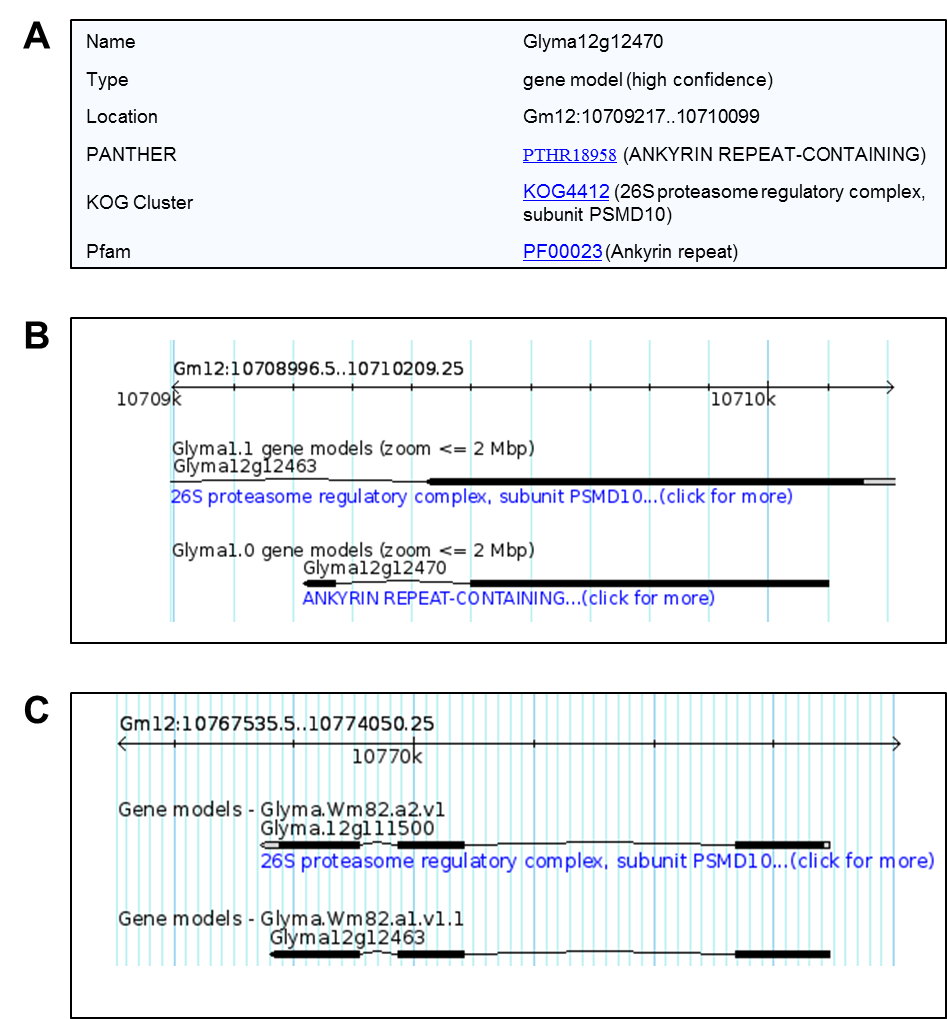


**S4 Fig. Screenshots of the *GmARP1* gene in the new and the old versions of genome sequence assemblies (**[**www.soybase.org**](http://www.soybase.org/)**).** (A) *Glyma12g12470* (Glyma.Wm82.a1.v1.1, Gmax1.01) cloned as *GmARP1* to generate transgenic soybean plants for this study. (B) Display of the *Glyma12g12470* (Glyma.Wm82.a1.v1.1, Gmax1.01) gene on soybean GBrowse used in this transgenic study. This Glyma 1.0 gene model was revised in the recent version (Glyma.Wm82.a2.v1) as *Glyma.12G111500* [see (C)]. (C) Recent version of the soybean genome assembly [Glyma.Wm82.a2.v1 (Gmax2.0)] displaying the *Glyma.12G111500* gene.
