# Supplementary material for: Tanscriptomic Study of the Soybean-Fusarium virguliforme Interaction Revealed a Novel Ankyrin-Repeat Containing Defense Gene, Expression of Whose during Infection Led to Enhanced Resistance to the Fungal Pathogen in Transgenic Soybean Plants
Source: PLoS One. 2016 Oct 19;11(10):e0163106. doi: 10.1371/journal.pone.0163106 (PMC5070833; doi:10.1371/journal.pone.0163106)
Supplement: S5 Fig — (DOCX) [file pone.0163106.s005.docx]

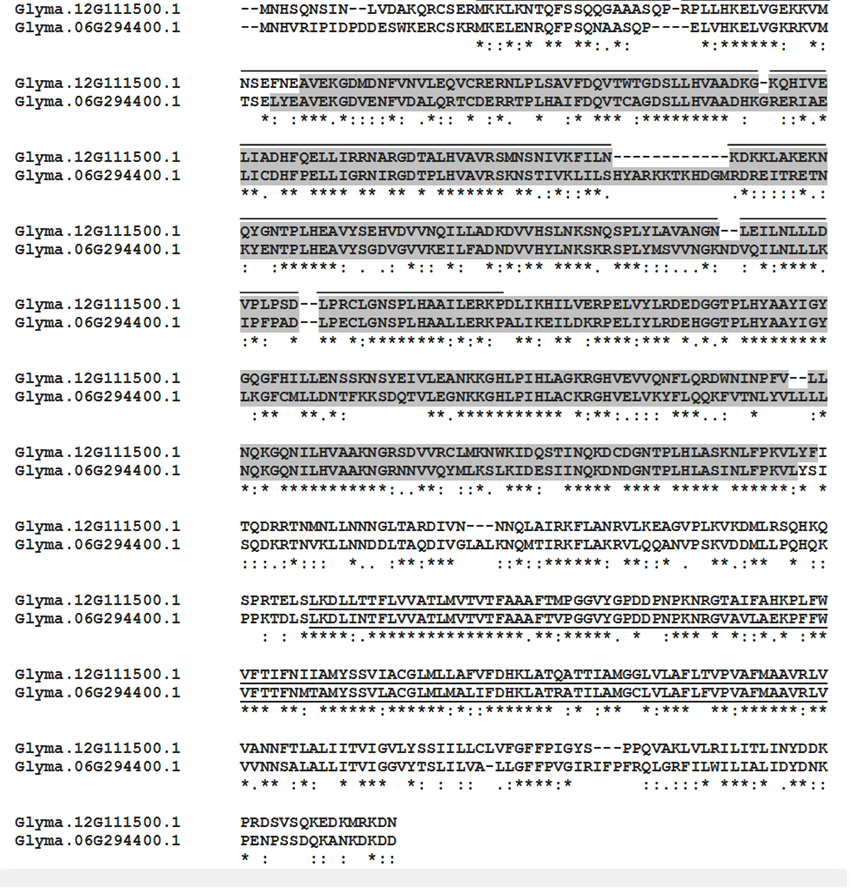


**S5 Fig. Sequence analysis of two ankyrin-repeat containing proteins downregulated following *F. virguliforme* infection.** Amino acid sequence alignment of ankyrin-repeat containing proteins encoded by *Glyma.12G111500.1* and its homolog *Glyma.06G294400* is presented. ‘*’, identical; ‘:’, conserved substitution, and ‘.’, semi-conserved substitution. *Glyma.12G111500.1* was predicted as *Glyma12g12470* (*GmARP1*) in the previous version of the soybean genome assembly (Glyma.Wm82.a1.v1.1 (Gmax1.01). The ankyrin-repeats are highlighted in grey and the *Glyma12g12470* (*GmARP1*) sequence used for transgenic studies is shown with a line over the sequence.
