# Supplementary material for: Tanscriptomic Study of the Soybean-Fusarium virguliforme Interaction Revealed a Novel Ankyrin-Repeat Containing Defense Gene, Expression of Whose during Infection Led to Enhanced Resistance to the Fungal Pathogen in Transgenic Soybean Plants
Source: PLoS One. 2016 Oct 19;11(10):e0163106. doi: 10.1371/journal.pone.0163106 (PMC5070833; doi:10.1371/journal.pone.0163106)
Supplement: S6 Fig — (DOCX) [file pone.0163106.s006.docx]

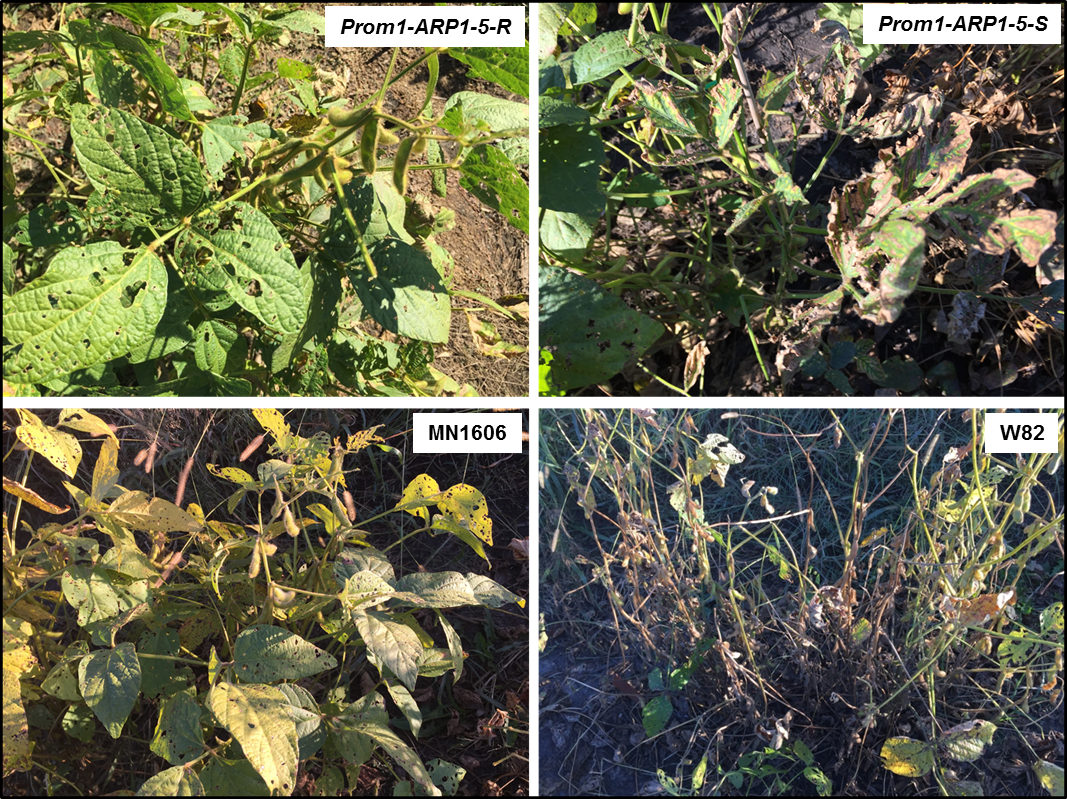


**S6 Fig. Phenotype of transgenic soybean plants carrying *GmARP1* fusion genes in a field trial.** *GmARP1* lines were resistant to *F. virguliforme*, whereas the recipient cultivar Williams 82 (W82) is susceptible the pathogen. Pictures were taken on October 7, 2015 (last date of scoring) when the plants were at maturity stage (R-stage) 6.0. Prom1-ARP1-5 R, an R_1_ resistant line of the Prom1-GmARP1-5 transformant; Prom1-ARP1-5 S*,* an R_1_ susceptible line of the Prom1-GmARP1-5 transformant. W82, Williams 82 is an SDS susceptible line, recipient of the transgene. MN1606, is an SDS resistant soybean cultivars.
