# Supplementary material for: Tanscriptomic Study of the Soybean-Fusarium virguliforme Interaction Revealed a Novel Ankyrin-Repeat Containing Defense Gene, Expression of Whose during Infection Led to Enhanced Resistance to the Fungal Pathogen in Transgenic Soybean Plants
Source: PLoS One. 2016 Oct 19;11(10):e0163106. doi: 10.1371/journal.pone.0163106 (PMC5070833; doi:10.1371/journal.pone.0163106)
Supplement: S7 Fig — (DOCX) [file pone.0163106.s007.docx]

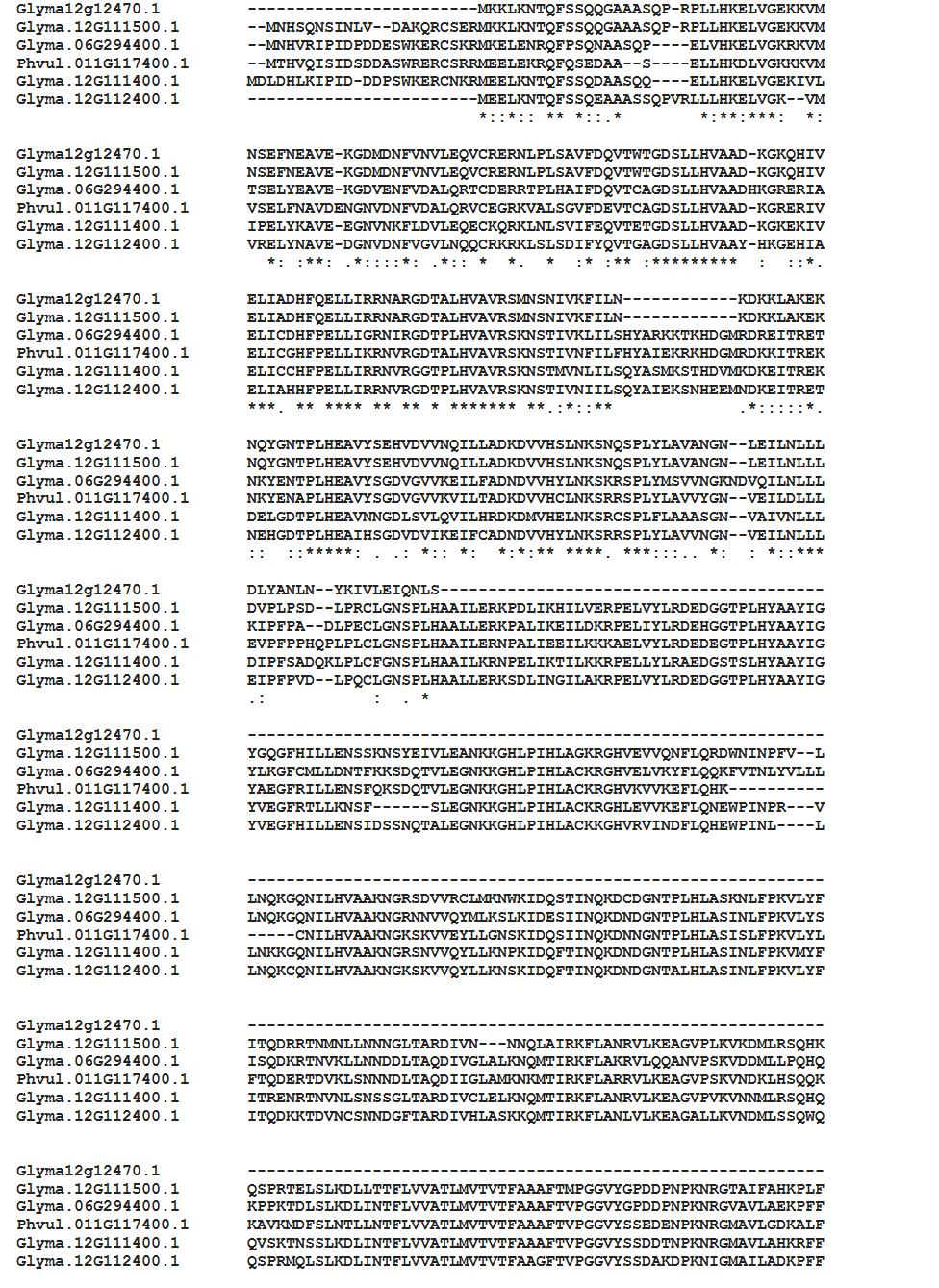


**S7 Fig. Sequence analysis of five ankyrin-repeat containing GmARP1 (*Glyma12g12470*) homologs.** Amino acid sequence alignment of GmARP1 and its five homologs. *, identical; :, conserved substitution, and ., semi-conserved substitution.
