# Supplementary material for: Tanscriptomic Study of the Soybean-Fusarium virguliforme Interaction Revealed a Novel Ankyrin-Repeat Containing Defense Gene, Expression of Whose during Infection Led to Enhanced Resistance to the Fungal Pathogen in Transgenic Soybean Plants
Source: PLoS One. 2016 Oct 19;11(10):e0163106. doi: 10.1371/journal.pone.0163106 (PMC5070833; doi:10.1371/journal.pone.0163106)
Supplement: S8 Fig — (DOCX) [file pone.0163106.s008.docx]

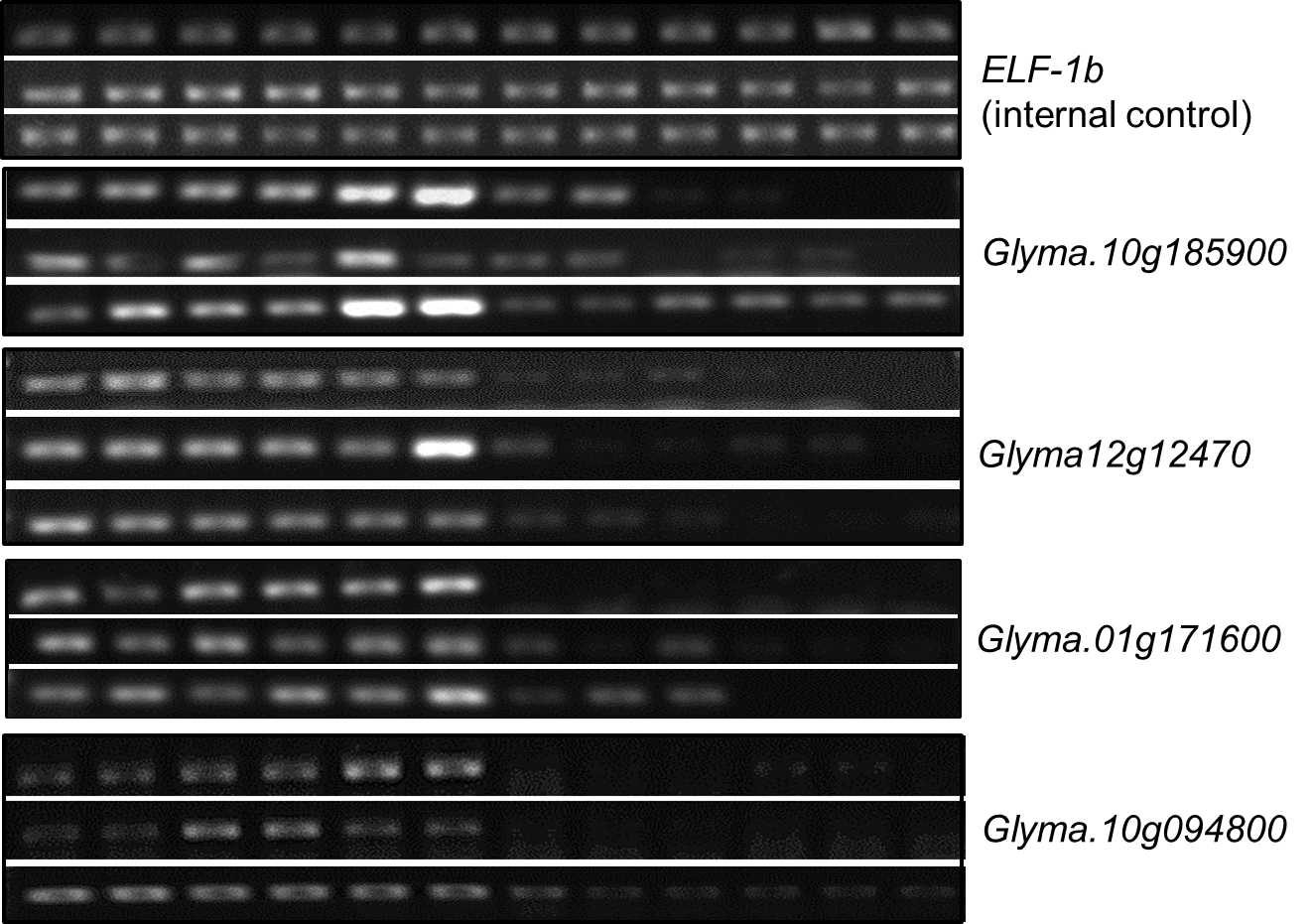


**S8 Fig. RT-PCR analysis of three independent replicates for the four selected genes.** Expression of the genes *Glyma.10g185900*, *Glyma12g12470*, *Glyma.01g171600*, and Glyma.*10g094800* were analyzed. Quantification of these data is presented in Figure 8C-8F.
