# Supplementary material for: Tanscriptomic Study of the Soybean-Fusarium virguliforme Interaction Revealed a Novel Ankyrin-Repeat Containing Defense Gene, Expression of Whose during Infection Led to Enhanced Resistance to the Fungal Pathogen in Transgenic Soybean Plants
Source: PLoS One. 2016 Oct 19;11(10):e0163106. doi: 10.1371/journal.pone.0163106 (PMC5070833; doi:10.1371/journal.pone.0163106)
Supplement: S1 Table — (DOCX) [file pone.0163106.s009.docx]

**S1 Table.** List of primers used for PCR and RT-PCR. Restriction endonuclease sites are highlighted.

| **Name of primer** | **Sequence (5’-3’)** | **Purpose** |
| --- | --- | --- |
| GmARP1-G-F | GAATTC***CCATATATCTGG***GGCGTTAATCCATTAACCTAAATCATGA | cloning GmARP1 in vector pTF102 |
| GmARP1-G-R | GAATTC***CCATATATCTGG***GTCTGATAATGGATGACCTGATAAGTCC | cloning GmARP1 in vector pTF102 |
| Prom1-F | CATGCAATGCACT***TCTAGA***TGTGGGAGTTTCAATGTG | cloning promoter 1 in vector pTF102 |
| Prom1-R | GAATTC***TCTAGA***CTATCAAGTATTTCAACGTTATTCAAC | cloning promoter 1 in vector pTF102 |
| Prom2-F | GAATTC***TCTAGA***GCCTAAGACATGGAGGGGAAAATAAATG | cloning promoter 2 in vector pTF102 |
| Prom2-R | GAATTC***TCTAGA***CCATATATCTGGCCACCAGCTTTGGTGTCATTGATA | cloning promoter 2 in vector pTF102 |
| Prom3-F | GAATTC***TCTAGA***CGGTCCCAACAACTAGTTTGCATTC | cloning promoter 3 in vector pTF102 |
| Prom3-R | GAATTC***TCTAGA***CCATATATCTGGGTCTGCACGCTTGTCAAAATATTAG | cloning promoter 3 in vector pTF102 |
| pTF102-ter-F | GAATTC***CCATATATCTGG***GGCGCACCATCGTCGGCTAC | cloning CaMV 35S terminator in vector pTF102 |
| pTF102-ter-R | TGCTCCACCATGTTGACCGGCATG | cloning CaMV 35S terminator in vector pTF102 |
| RT-pTF102-R | GTAGCCGACGATGGTGCGCC | characterization of transgenic lines |
| GmARP1-RT-F | CTGGACTGGTGATTCGTTGCTTC | characterization of transgenic lines |
| GmARP1-RT-R | ACGGTGATTGGTTTGACTTGTTC | characterization of transgenic lines |
| Elf1b-F | CGCTCAAGGGGTAAGATTCA | characterization of transgenic lines |
| Elf1b-R | CCCACAATAAACCAGGCATC | characterization of transgenic lines |
