# Supplementary material for: Tanscriptomic Study of the Soybean-Fusarium virguliforme Interaction Revealed a Novel Ankyrin-Repeat Containing Defense Gene, Expression of Whose during Infection Led to Enhanced Resistance to the Fungal Pathogen in Transgenic Soybean Plants
Source: PLoS One. 2016 Oct 19;11(10):e0163106. doi: 10.1371/journal.pone.0163106 (PMC5070833; doi:10.1371/journal.pone.0163106)
Supplement: S2 Table — (DOCX) [file pone.0163106.s010.docx]

**S2 Table**. Number of plants resistant to Basta for *GmARP1* transgenic lines used in the field trial.

| **Lines** | **# Resistant** | **# Susceptible** | **Total** |
| --- | --- | --- | --- |
| *Prom1-ARP1-5* | 16 | 5 | 21 |
| *Prom2-ARP1-3* | 13 | 0 | 13 |
| *Prom2-ARP1-7* | 33 | 9 | 42 |
| *Prom2-ARP1-9* | 26 | 5 | 31 |
| *Prom3-ARP1-3* | 4 | 7 | 11 |
| *Prom3-ARP1-11* | 17 | 4 | 21 |
