# Supplementary material for: Tanscriptomic Study of the Soybean-Fusarium virguliforme Interaction Revealed a Novel Ankyrin-Repeat Containing Defense Gene, Expression of Whose during Infection Led to Enhanced Resistance to the Fungal Pathogen in Transgenic Soybean Plants
Source: PLoS One. 2016 Oct 19;11(10):e0163106. doi: 10.1371/journal.pone.0163106 (PMC5070833; doi:10.1371/journal.pone.0163106)
Supplement: S3 Table — (DOCX) [file pone.0163106.s011.docx]

**S3 Table**. Method of scoring SDS in the field ([www.siu.edu/~soybean](http://www.siu.edu/~soybean)).

| **Score** | **Description of Symptoms** |
| --- | --- |
| 1 | 1-10% of leaf surface chlorotic, OR 1-5% necrotic |
| 2 | 10-20% of leaf surface chlorotic, OR 6-10% necrotic |
| 3 | 20-40% of leaf surface chlorotic, OR 11-20% necrotic |
| 4 | 40-60% of leaf surface chlorotic, OR 21-40% necrotic |
| 5 | Greater than 60% of leaf surface chlorotic, OR greater than 40% necrotic |
| 6 | Premature leaf drop up to 1/3 defoliation |
| 7 | Premature leaf drop up to 2/3 defoliation |
| 8 | Premature leaf drop greater than 2/3 defoliation |
| 9 | Premature death |
