# Supplementary material for: Tanscriptomic Study of the Soybean-Fusarium virguliforme Interaction Revealed a Novel Ankyrin-Repeat Containing Defense Gene, Expression of Whose during Infection Led to Enhanced Resistance to the Fungal Pathogen in Transgenic Soybean Plants
Source: PLoS One. 2016 Oct 19;11(10):e0163106. doi: 10.1371/journal.pone.0163106 (PMC5070833; doi:10.1371/journal.pone.0163106)
Supplement: S4 Table — (DOCX) [file pone.0163106.s012.docx]

**S4 Table**. List of primers used for RT-PCR of four selected genes.

| **Name of primer** | **Sequence (5'-3')** | **Amplicon size (bp)** |
| --- | --- | --- |
| Glyma10g32980-Fw | GTGGACTCTCTTTTCACCCTTGT | 388 |
| Glyma10g32980-Rev | GAAGTGCTGCTTGCCTTGTG |  |
| Glyma10g12400-Fw | TGTGGAAATGGCACCTTATG | 153 |
| Glyma10g12400-Rev | ACAGGCAAATCAAACCCAGT |  |
| Glyma01g37680-Fw | ATCATCAGTCACCAGAATACCAGAT | 360 |
| Glyma01g37680-Rev | GACACTTCAAAAACAACGAGAAATC |  |
| Glyma12g12470-Fw | CTGGACTGGTGATTCGTTGCTTC | 326 |
| Glyma12g12470-Rev | ACGGTGATTGGTTTGACTTGTTC |  |
| Elf1b-F | CGCTCAAGGGGTAAGATTCA | 500 |
| Elf1b-R | CCCACAATAAACCAGGCATC |  |
